# Competition between VanU_G_ Repressor and VanR_G_ Activator Leads to Rheostatic Control of *vanG* Vancomycin Resistance Operon Expression

**DOI:** 10.1371/journal.pgen.1005170

**Published:** 2015-04-21

**Authors:** Florence Depardieu, Vincent Mejean, Patrice Courvalin

**Affiliations:** 1 Unité des Agents Antibactériens, Institut Pasteur, Paris, France; 2 Laboratoire de Bioénergétique et Ingénierie des protéines, Aix Marseille Université, Marseille, France; Indiana University, UNITED STATES

## Abstract

*Enterococcus faecalis* BM4518 is resistant to vancomycin by synthesis of peptidoglycan precursors ending in D-alanyl-D-serine. In the chromosomal *vanG* locus, transcription of the resistance genes from the *P_YG_* resistance promoter is inducible and, upstream from these genes, there is an unusual three-component regulatory system encoded by the *vanURS_G_* operon from the *P_UG_* regulatory promoter. In contrast to the other *van* operons in enterococci, the *vanG* operon possesses the additional *vanU_G_* gene which encodes a transcriptional regulator whose role remains unknown. We show by DNase I footprinting, RT-qPCR, and reporter proteins activities that VanU_G_, but not VanR_G_, binds to *P_UG_* and negatively autoregulates the *vanURS_G_* operon and that it also represses *PYG* where it overlaps with VanR_G_ for binding. In clinical isolate BM4518, the transcription level of the resistance genes was dependent on vancomycin concentration whereas, in a *ΔvanUG* mutant, resistance was expressed at a maximum level even at low concentrations of the inducer. The binding competition between VanU_G_ and VanR_G_ on the *P_YG_* resistance promoter allowed rheostatic activation of the resistance operon depending likely on the level of VanR_G_ phosphorylation by the VanS_G_ sensor. In addition, there was cross-talk between VanS_G_ and VanR'_G_, a VanR_G_ homolog, encoded elsewhere in the chromosome indicating a sophisticated and subtle regulation of vancomycin resistance expression by a complex two-component system.

## Author Summary

Various modes of gene regulation coexist in cells. One corresponds to the “switch on/ off” mechanism in which the regulator induces the promoter to a defined level. In another mechanism, the regulator activates the promoter to various levels according to the intensity or the nature of an input signal. In this study, we show that in VanG-type vancomycin resistant *Enterococcus faecalis* a repressor (VanU_G_) allows rheostatic expression of a target resistance promoter by competing with a response regulator (VanR_G_) which otherwise acts together with a sensor (VanS_G_) by a "switch on/off" mechanism as part of a two-component regulatory system. Unusually, both regulators are encoded in the same operon.

## Introduction

Vancomycin-resistant enterococci are a major cause of nosocomial infections and an important public health problem because the treatment options for the infections they cause are very limited [1]. Vancomycin, which can be the only antibiotic effective against multiresistant clinical isolates, acts by binding to the C-terminal D-alanyl-D-alanine (D-Ala-D-Ala) residues of peptidoglycan precursors blocking the extracellular steps in peptidoglycan synthesis [2]. Resistance in *Enterococcus* is mediated by nine types of operons that produce modified peptidoglycan precursors ending in D-Ala-D-Lac (*vanA*, *-B*, *-D*, and-*M*) or D-Ala-D-Ser (*vanC*, -*E*, -*G*, -*L*, and-*N*) to which vancomycin bind with a low affinity and from the elimination of the high affinity precursors ending in D-Ala-D-Ala [3–6].

Expression of the vancomycin resistance operons is regulated by VanS/VanR-type two-component signal transduction systems composed of a membrane-bound histidine kinase (VanS-type) and a cytoplasmic response regulator (VanR-type) that acts as a transcriptional activator [3]. The sensors modulate the levels of phosphorylation of the regulators. In the presence of vancomycin, VanS acts primarily as a kinase that autophosphorylates and transfers its phosphate to VanR. Phosphorylated VanR binds to the promoters upstream from the *vanRS* regulatory and resistance operons leading to increased transcription of the regulatory and resistance genes [7–9]. The phosphatase activity of VanS-type sensors is required for negative regulation of the resistance genes in the absence of vancomycin preventing accumulation of VanR-type regulators phosphorylated by acetylphosphate or by kinases encoded by the host chromosome [7, 10].

**Fig 1:**
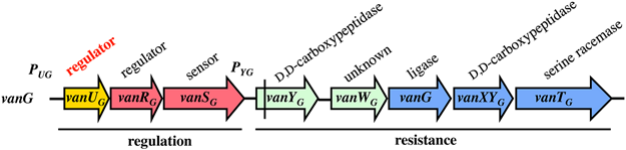
Schematic representation of the *vanG* operon. Open arrows represent coding sequences and indicate direction of transcription. The regulatory genes are in red, the resistance genes in blue and accessory genes in green. The additional regulatory gene, *vanUG*, is in yellow. The vertical bar in *vanYG* indicates a frameshift mutation leading to a truncated protein.

VanG-type *Enterococcusfaecalis* clinical isolates from Australia and Canada are distinct from other Van-type enterococci. The chromosomal *vanG* cluster (Fig 1) confers resistance to vancomycin (MICs, 16 μg/ml) by inducible synthesis of precursors ending in D-Ala-D-Ser [11]. It contains the *vanY*_*G*_,*W*_*G*_,*G*,*XY*_*G*_,*T*_*G*_ resistance genes, the last three strictly required for resistance encode, respectively, a VanG ligase to synthesize D-Ala-D-Ser, a VanXY_G_ D,D-carboxypeptidase to hydrolyse D-Ala-D-Ala, and a VanT_G_ membrane bound serine racemase to produce D-Ser (Fig 1). As opposed to the other *van* gene clusters, the *vanG* regulatory operon contains three genes, *vanU*_*G*_, *vanR*_*G*_, and *vanS*_*G*_, encoding a "three component" regulatory system (Fig 1). Additional gene *vanU*_*G*_ encodes a transcriptional regulator belonging to the Xre protein family and of unknown function. The *vanURS*_*G*_ genes are co-transcribed, even in the absence of vancomycin, from the *P*_*UG*_ regulatory promoter, whereas transcription of the resistance genes is inducible and initiated from the *P*_*YG*_ resistance promoter [11].

Cryptic *vanG*-like operons are common in *Clostridium difficile*, a major human pathogen which is a target for vancomycin, and a *vanU*_*G*_ gene encoding a protein identical to VanU_G_ was found in a clinical isolate (GenBank N° AVLW01000050). A VanU_G_-like protein (GenBank N° YP002939420), 79% identical with VanU_G_, was detected in an *Eubacterium* associated with a two-component system controlling an ABC-type transporter and a protein (GenBank N°YP007781704) with 76% identity was reported in *Ruminococcus bromii* associated with a CheY related regulator and a partial *vanG* operon. These regulators have not been studied.

We report the role of VanU_G_ in the transcription of the *vanG* operon in *E*.*faecalis*. We show that VanU_G_ binds to the *P*_*UG*_ regulatory and *P*_*YG*_ resistance promoters and negatively regulates the *vanURS*_*G*_ regulatory and resistance operons. In contrast, VanR_G_ binds only to *P*_*YG*_. It thus appears that, upon induction by vancomycin, the VanS_G_ sensor phosphorylates VanR_G_ which competes and displaces VanU_G_ from *P*_*YG*_ leading to transcription of the resistance operon in a dose dependent manner. Thus, rheostatic regulation of resistance gene expression results from binding of a repressor and an activator encoded in a single operon to the same promoter.

## Results

### VanU_G_ but not VanR_G_ binds to the *P*_*UG*_ regulatory promoter

**Fig 2:**
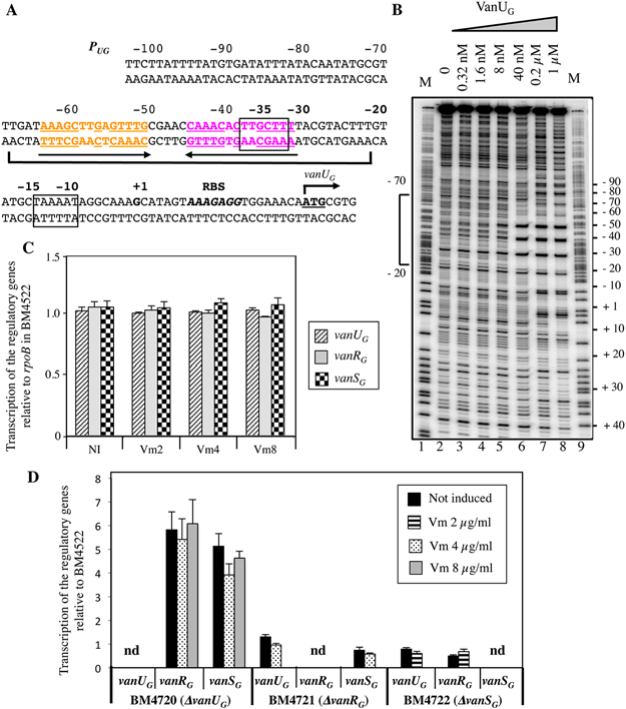
Binding sites of VanU_G_ to the *P*_*UG*_ regulatory promoter (A, B) and regulatory genes transcription (C, D). (A) Sequence of the *P*_*UG*_ region. The transcriptional start site (+1) is in boldface and the -35 and -10 sequences are boxed. The translational start site is in boldface and underlined and the ribosome binding site (RBS) is in boldface and in italics. Regions protected from DNase I cleavage by VanU_G_ are delineated by a bracket. The binding motif is composed of two 14-bp imperfect inverted repeats indicated in orange and purple and by arrows; the complementary bases are underlined. (B) DNase I footprinting analysis of the binding of VanU_G_ to *P*_*UG*_. A 357-bp DNA fragment was amplified from the *P*_*UG*_ promoter region using a labeled reverse primer (VanG126) to radiolabel the template strand. Increasing amounts of VanU_G_, indicated above each lane, were incubated with the DNA probe. The bracket indicates the region protected from DNase I cleavage by VanU_G_ and the co-ordinates of protection relative to the transcriptional start site are indicated on the left. M is the A+G Maxam and Gilbert sequencing reaction lane of the probe used as a size marker and the nucleotide positions are indicated at the right. Transcription of the regulatory genes by RT-qPCR in transconjugant BM4522 (C) and deletant derivatives relative to the same genes of BM4522 (D). The strains are indicated at the bottom. Results are presented in arbitrary units normalized to the *rpoB* transcripts in the same strain and in BM4522 under similar conditions. Each strain, not induced or induced by vancomycin, was tested in triplicate in two independent experiments. The bars represent the means and the error bars the standard deviations; nd, not detectable. NI, not induced. Vm, vancomycin.

Primer extension of the region upstream from *vanU*_*G*_ indicated that, irrespective of induction, the transcriptional start site for *vanURS*_*G*_ was located 22 bp upstream from the translation initiation codon of *vanU*_*G*_ [11]. The *P*_*UG*_ promoter consists of -35 and -10 regions corresponding to δ70 recognition sequences separated by 17 bp (Fig 2A). To determine if VanU_G_ and VanR_G_ bind to the *P*_*UG*_ regulatory promoter region and to identify putative specific binding sites, DNaseI footprinting experiments were carried out. A radiolabeled PCR probe corresponding to positions -247 to +110 relative to the transcription initiation site of *P*_*UG*_ was incubated with increasing amounts of purified VanU_G_, VanR_G_, and VanR_G_ phosphorylated (VanR_G_-P) by acetyl phosphate. The *P*_*UG*_ region protected by VanU_G_ depended on the protein concentration, extending from -70 to -20 (positions relative to the transcription initiation site) overlapping the -35 sequence at a low concentration (Fig 2B, lane 6) and from -70 to +10 at higher concentrations (Fig 2B, lanes 7 and 8). The region (-70 to -20) contained two adjacent imperfect palindromic sequences likely corresponding to the binding motifs of VanU_G_ (Fig 2A). As opposed to the wild-type fragment, two DNA fragments containing double mutations in the imperfect dyad symmetry operator of *P*_*UG*_ were not retarded by VanU_G_, indicating a key role in VanU_G_ binding (S1 Fig). The appearance of several DNase I hypersensitive sites (Fig 2B) corresponding to bending of the DNA duplex suggested binding of two VanU_G_ monomers or dimers. This is consistent with the presence of two inverted repeats in the *P*_*UG*_ region (Fig 2A) and with the two-step gel retardation (S1 Fig). In contrast to VanU_G_, VanR_G_ and VanR_G_-P did not bind to the *P*_*UG*_ promoter.

### VanU_G_ acts as a repressor of the *P*_*UG*_ regulatory promoter

The *vanG* operon is part of a large genetic element and is transferable from *E*. *faecalis* BM4518 to *E*. *faecalis* JH2-2 from chromosome to chromosome [11]. Since clinical isolate BM4518 is not transformable, we studied the VanURS_G_ system in transconjugant BM4522 (JH2-2::*vanG*) (S1 Table). To determine the role of VanU_G_ on *P*_*UG*_, the *vanU*_*G*_, *vanR*_*G*_, and *vanS*_*G*_ genes of BM4522 were inactivated individually by in-frame deletions leading to BM4720(Δ*vanU*_G_), BM4721(Δ*vanR*_G_), and BM4722(Δ*vanS*_G_). Transcription of the regulatory genes was quantified by RT-qPCR. In BM4522, low level transcription occured at similar levels without and with various concentrations of vancomycin indicating that the *P*_*UG*_ promoter was not inducible by vancomycin (Fig 2C). In the absence of *vanU*_*G*_, *vanR*_*G*_ and *vanS*_*G*_ were transcribed in the absence or presence of vancomycin at higher level (≈ 5-fold) from *P*_*UG*_ indicating that VanU_G_ acted as a repressor on this promoter region (Fig 2D). In the absence of *vanR*_*G*_ or *vanS*_*G*_, transcription of the regulatory genes remained unchanged even in the presence of vancomycin.

To confirm regulation of *P*_*UG*_ by VanU_G_, the *vanURS*_*G*_ genes were cloned into vancomycin susceptible *Escherichia coli* NR698 [12] under the control of promoter *P*_*spank*_ upstream from *P*_*UG*_ fused to a chloramphenicol acetyltransferase (CAT) reporter gene, the two promoters being separated by a transcription terminator (*term*) (Table 1). Subsequently, each of the three genes was inactivated. *E*. *coli* RNA polymerase bound to the *P*_*UG*_ promoter (S2A Fig) which was active in the new host, in the presence or in the absence of vancomycin (Table 1). CAT was produced at a maximum level in the absence of *vanU*_*G*_ by plasmids pAT952(*P*_*spank*_ *termP*_*UG*_ *cat*), pAT966(*P*_*spank*_ *vanR*_*G*_ *termP*_*UG*_ *cat*), and pAT969(*P*_*spank*_ *vanRS*_*G*_ *termP*_*UG*_ *cat*) (Table 1). In contrast, in the presence of VanU_G_, CAT production was decreased to similar basal levels by plasmids pAT965(*P*_*spank*_ *vanU*_*G*_ *termP*_*UG*_ *cat*), pAT967(*P*_*spank*_ *vanUR*_*G*_ *termP*_*UG*_ *cat*), and pAT968 (*P*_*spank*_ *vanURS*_*G*_ *termP*_*UG*_ *cat*) (Table 1). These results confirmed that VanU_G_ acts as a strong repressor on the *P*_*UG*_ promoter.

**Table 1:** CAT specific activities of *P*_*UG*_ promoter in *E*. *coli* NR698.

	CAT specific activity^a^	
Plasmid	Uninduced	Vancomycin
pDR111 (*P*_*spank*_)^b^	8 ± 4	11 ± 5
pAT949 (*P*_*spank*_ *cat*)	360 ± 13	406 ± 22
pAT950 (*P*_*spank*_ *termcat*)^c^	80 ± 1	91 ± 5
pAT964 (*P*_*spank*_ *vanU*_*G*_ *termcat*)	65 ± 5	64 ± 6
pAT952 (*P*_*spank*_ *termP*_*UG*_ *cat*)	2023 ± 196	2156 ± 105
pAT965 (*P*_*spank*_ *vanU*_*G*_ *termP*_*UG*_ *cat*)	134 ± 15	172 ± 12
pAT966 (*P*_*spank*_ *vanR*_*G*_ *termP*_*UG*_ *cat*)	1856 ± 125	2064 ± 269
pAT967 (*P*_*spank*_ *vanUR*_*G*_ *termP*_*UG*_ *cat*)	159 ± 13	146 ± 14
pAT968 (*P*_*spank*_ *vanURS*_*G*_ *termP*_*UG*_ *cat*)	115 ± 13	109 ± 13
pAT969 (*P*_*spank*_ *vanRS*_*G*_ *termP*_*UG*_ *cat*)	1557 ± 64	1478 ± 100

^a^ Results are expressed in nanomoles of product formed per minute and per milligram of protein in S100 extracts. Induction was performed with vancomycin (0.25 μg/ml). Data are means ± standard deviation obtained from a minimum of three independent extracts.

^b^ The *P*_*spank*_ promoter is constitutive due to low expression in the absence of induction by IPTG.

^C^ *term* corresponds to the T4 transcription terminator.

### The VanR_G_S_G_ two-component system is functional

**Fig 3:**
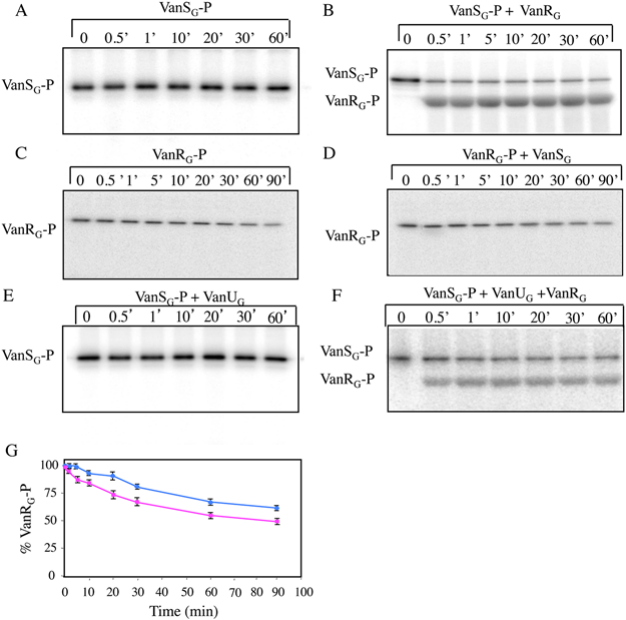
Autophosphorylation of VanS_G_ (A), phosphotransfer from VanS_G_-P to VanR_G_ (B), phosphorylation of VanR_G_ by acetyl [^32^P] phosphate (C), hydrolysis of VanR_G_-P by VanS_G_ (D), and phosphotransfer from VanS_G_ to VanU_G_ (E) or to VanU_G_ plus VanR_G_ (F). Quantitative analysis of phosphorylated VanR_G_ in panels C and D (G). (A) Purified VanS_G_ was incubated with [γ-^32^P]-ATP for 1 h at room temperature to test autophosphorylation. (B) After autophosphorylation of VanS_G_ (time 0), purified VanR_G_ was added, samples were removed at the indicated times (in min), mixed with β-mercaptoethanol stop solution on ice and separated by SDS-PAGE (15%). Transfer of radioactivity to VanR_G_ was revealed by autoradiography. (C) Purified VanR_G_ was incubated with acetyl[^32^P]phosphate for 1 h at room temperature (time0), excess acetyl[^32^P]phosphate was removed by using a Sephadex G-50 Quick-Spin column, and phosphorylated VanR_G_ was incubated at room temperature either alone or (D) following the addition of purified VanS_G_. Samples were removed at the indicated times (in min), mixed with β-mercaptoethanol-stop solution on ice, resolved by SDS-PAGE (15%), and subjected to autoradiography. After autophosphorylation of VanS_G_ (time 0), purified VanU_G_ was added alone (E) or with VanR_G_ (F), samples were removed at the indicated times (in min), mixed with β-mercaptoethanol stop solution on ice and separated by SDS-PAGE (12%). Transfer of radioactivity to VanR_G_ but not to VanU_G_ was revealed by autoradiography. (G) Hydrolysis in the absence (blue line, panel C) or in the presence (pink line, panel D) of VanS_G_ of purified VanR_G_ labeled with acetyl[^32^P]phosphate was detected on a phosphor storage screen and percent quantified. Results are the means of four independent experiments and the bars indicate standard deviations.

Transcription of the resistance genes is under the control of VanURS_G_ and, as discussed above, VanU_G_ negatively autoregulates *vanURS*_*G*_ transcription from the *P*_*UG*_ regulatory promoter. To determine if VanR_G_ and VanS_G_ acted as a two-component system and to study the putative interaction of VanU_G_ with these proteins, VanU_G_, VanR_G_, and the cytoplasmic histidine kinase domain of VanS_G_ were purified as C-terminal His-tag proteins (S1 Table). VanS_G_ autophosphorylated in the presence of [γ-^32^P]-ATP (Fig 3A). When incubated with purified VanU_G_ or VanR_G_, phosphorylated VanS_G_ transferred its phosphate group to VanR_G_ (Fig 3B) but not to VanU_G_ (Fig 3E). Phosphorylation of VanR_G_ was fast and efficient, occurring in less than a minute. To test the phosphatase activity of VanS_G_, hydrolysis of VanR_G_-P over time was analysed in the absence or in the presence of VanS_G_. Purified [^32^P]-VanR_G_ was stable in vitro for at least 30min and then dephosphorylated slowly (Fig 3C); addition of purified VanS_G_ increased dephosphorylation only slightly (Fig 3D–3G). These results indicate that VanRS_G_ was functional and had characteristics similar to those of other VanRS-type two-component systems [7, 9] and that VanU_G_ did not affect phosphorylation nor dephosphorylation of VanR_G_ and VanS_G_ (Fig 3E and 3F).

### VanU_G_ and VanR_G_ bind to overlapping sites of the *P*_*YG*_ resistance promoter

**Fig 4:**
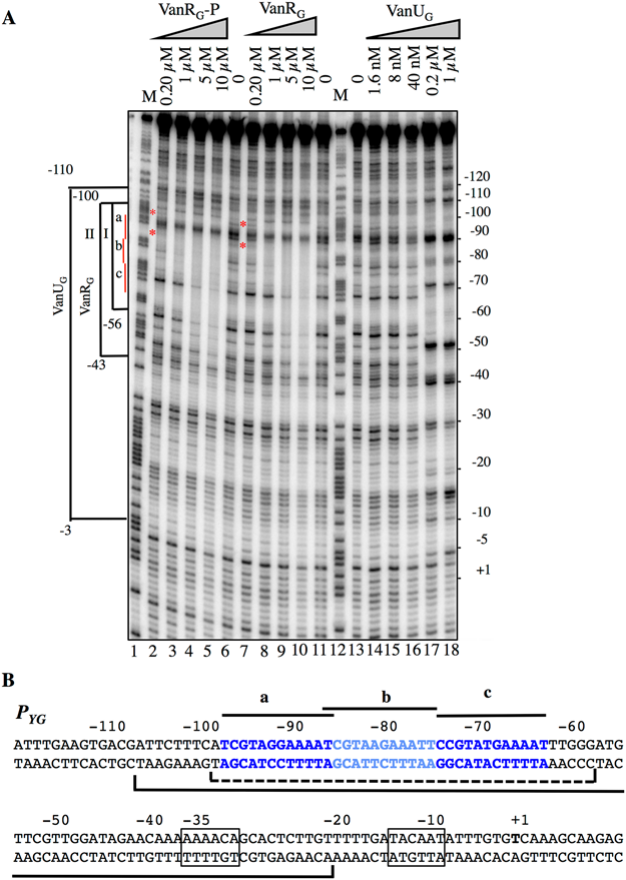
Binding of VanU_G_, VanR_G_, and VanR_G_-P to the *P_YG_* resistance promoter. (A) DNase I footprinting analysis. A 233-bp DNA fragment was amplified from the *PYG* promoter region using a labeled reverse primer (YG10) to radiolabel the template strand. Increasing amounts of VanU_G_, VanR_G_, or VanR_G_-P, indicated at the top, were incubated with the DNA probe. The brackets indicate the regions protected from DNase I cleavage by VanU_G_, VanR_G_, or VanR_G_-P, and the co-ordinates of protection relative to the transcriptional start site are indicated on the left. The three 12-bp VanR_G_ binding sites (a, b, c) are indicated in red on the left. The red asteriks indicate the slight difference in affinity in favor of VanR_G_-P (lane 2) in comparison with VanR_G_ (lane 7), both at 0.2 μM. M is the A+G Maxam and Gilbert sequencing reaction lane of the probe used as a size marker and the nucleotide positions are indicated at the right. (B) Sequence of the *PYG* promoter region. The transcriptional start site (+1) is in boldface and the -35 and -10 sequences are boxed. The three (a, b, c) 12-bp putative VanR_G_ binding sites are in blue and indicated by black lines. The region protected from DNase I cleavage by VanU_G_ is delineated by a black bracket and that of VanR_G_ or VanR_G_-P is delineated by a dotted bracket.

To study the putative binding of VanU_G_ and VanR_G_ to the *P*_*YG*_ region and to identify specific binding sites, DNaseI footprinting experiments were carried out. The inducible *P*_*YG*_ promoter is composed of -35 (AAAACA) and -10 (TACAAT) regions separated by 16 bp which have similarity with δ70 recognition sequences, although the -35 sequence is not conserved consistent with the fact that the promoter is positively regulated (Fig 4B). Analysis of the *P*_*YG*_ region revealed three 12-bp directly repeated VanR_G_ binding motifs and a deduced consensus sequence (T/C)CGTANGAAA(T/A)T was analogous to that in the *P*_*R*_ and *P*_*H*_ *vanA* operon promoters [13]. In the *P*_*UG*_ region, similar sequences were not found (Fig 2A) which could explain lack of VanR_G_ binding. The radiolabeled probe corresponding to positions -163 to +69 relative to the transcription initiation point of the *P*_*YG*_ promoter and containing the three conserved sequences was incubated with increasing amounts of purified VanU_G_, VanR_G_, and VanR_G_-P (Fig 4). The three proteins protected in a concentration-dependent manner an overlapping DNA region that included the three direct repeats. The *P*_*YG*_ region protected by VanU_G_ was much larger than that by VanR_G_ and VanR_G_-P extending from -110 to -3 and overlapped the -35 sequence at 0.2 and 1μM (Fig 4A, lanes 17 and 18). The *P*_*YG*_ region protected by VanR_G_ and VanR_G_-P extended from -100 to -56 at low concentration (Fig 4A, bracket I, lanes 3 and 8) and from -100 to -43 at higher concentrations (Fig 4A, bracket II, lanes 4 and 5, and 9 and 10). There were three binding motifs a, b, and c with different affinities for VanR_G_ and VanR_G_-P in the *P*_*YG*_ promoter region (Fig 4). Only a slight difference in affinity in favor of VanR_G_-P at 0.2μM was noted for the "a" site (Fig 4A, lane 2) compared with VanR_G_ which could be due to inefficient phosphorylation of VanR_G_ by acetylphosphate. VanR_G_ and VanR_G_-P bound to the a and b sites (Fig 4A, lanes 2, 3, and 8) with higher affinity than to the c site (Fig 4A, lanes 4 and 5, and 9 and 10), whereas VanU_G_ bound to this DNA region with the same affinity (Fig 4A).

### VanU_G_ allows rheostatic expression of the resistance genes

**Fig 5:**
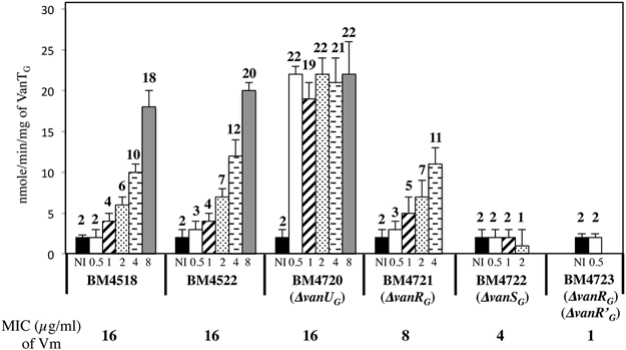
VanT_G_ racemase specific activity in membrane extracts from clinical isolate BM4518, transconjugant BM4522, and its deletant derivatives. Vancomycin (Vm) inducing concentrations (μg/ml) and MICs are indicated at the bottom. NI, not induced. The error bars represent the standard deviations from at least three independent experiments (eight for BM4723) and the values above the bars are the means of specific activity defined as the number of nanomoles of product formed at 37°C per minute per milligram of protein contained in the extracts.

To study the consequences of the binding of VanU_G_ and VanR_G_ to overlapping regions of *P*_*YG*_ on the expression of the resistance genes, the VanT_G_ serine racemase was used as a reporter (Fig 5). In clinical isolate BM4518 and transconjugant BM4522, synthesis of the serine racemase was dependent on the concentration of vancomycin (Fig 5). In contrast, in BM4720(Δ*vanU*_*G*_), the resistance operon was expressed at its maximum even at low concentrations of vancomycin. These results suggested that VanU_G_ acts as a repressor of *P*_*YG*_ and that, in its absence, there is no fine-tuning of resistance expression from this promoter. Thus, modulation of transcription by vancomycin was due to the phosphorylation level of VanR_G_ mediated by VanS_G_ provided that VanU_G_ was present. Surprisingly, as in the wild-type strain, induction was dependent on the concentration of the inducer in BM4721(Δ*vanR*_*G*_) (Fig 5). This could be accounted for by the presence of a VanR homolog in the host. In fact, we found, in both *E*.*faecalis* BM4518 and transconjugant BM4522 which were entirely sequenced (GenBank N°PRJNA245745), a gene specifying a VanR'_G_ protein with 65% identity with VanR_G_ (S3A Fig). In BM4722(Δ*vanS*_*G*_) there was no synthesis of VanT_G_ in the presence of vancomycin indicating that VanR_G_ and VanR'_G_ are not phosphorylated in the absence of VanS_G_. Double mutant BM4723(Δ*vanR*_*G*_, Δ*vanR'*_*G*_) derived from *E*. *faecalis* BM4721(Δ*vanR*_*G*_) was susceptible to vancomycin (MIC, 1μg/ml) and VanT_G_ production was no longer inducible by vancomycin, indicating cross-talk between VanS_G_ and VanR'_G_ (Fig 5). To avoid interference by this regulator, transcription from the *P*_*YG*_ promoter was studied in *E*.*coli* NR698 since *E*. *coli* RNA polymerase was able to bind to this promoter (S2B Fig). The *vanURS*_*G*_, *vanRS*_*G*_, and *vanUS*_*G*_ genes were cloned under the control of *P*_*spank*_ upstream from the *P*_*YG*_ transcriptionally fused to a *cat* gene generating pAT970 (*P*_*spank*_ *vanURS*_*G*_ *termP*_*YG*_ *cat*), pAT971 (*P*_*spank*_ *vanRS*_*G*_ *termP*_*YG*_ *cat*), and pAT972 (*P*_*spank*_ *vanUS*_*G*_ *termP*_*YG*_ *cat*). In the absence of VanU_G_, induction by vancomycin led to similar levels of CAT synthesis in the strain harboring pAT971 (*P*_*spank*_ *vanRS*_*G*_ *termP*_*YG*_ *cat*) whatever the concentration of the inducer, whereas with pAT970 (*P*_*spank*_ *vanURS*_*G*_ *termP*_*YG*_ *cat*) CAT production depended on the vancomycin concentration (Table 2). These results confirmed that, in the presence of vancomycin, VanU_G_ is required for rheostatic gene transcription from *P*_*YG*_ and that VanR_G_ phosphorylation is essential for expression of the resistance genes since, in the absence of this regulator in pAT972 (*P*_*spank*_ *vanUS*_*G*_ *termP*_*YG*_ *cat*), the level of CAT activity was low, both without (74U±9) and with (104 U ± 13) vancomycin (0.30 μg/ml). In the absence of vancomycin, CAT activity was lower in *E*. *coli* producing *vanU*_*G*_ encoded by pAT970 (*P*_*spank*_ *vanURS*_*G*_ *termP*_*YG*_ *cat*) than in its counterpart harboring pAT971 (*P*_*spank*_ *vanRS*_*G*_ *termP*_*YG*_ *cat*). This confirms that VanU_G_ acts as a repressor on the *P*_*YG*_ resistance promoter (Table 2).

**Table 2:** CAT specific activities of *P_YG_* promoter in *E*. *coli* NR698.

Plasmid		Vancomycin		
	0	0.2	0.3	0.4
pAT970 (*P*_*spank*_ *vanURS*_*G*_ *termP*_*YG*_ *cat*)	264 ± 23^a^	566 ± 54	797 ± 64	1283 ± 118
pAT971 (*P*_*spank*_ *vanRS*_*G*_ *termP*_*YG*_ *cat*)	544 ± 48	1585 ± 115	1556 ± 162	1487 ± 142

^a^ Results are expressed in nanomoles of product formed per minute and per milligram of protein in cytoplasmic extracts. Data are means ± standard deviation obtained from a minimum of three independent extracts.

^b^ The *P*_*spank*_ promoter is constitutive due to low expression in the absence of induction by IPTG.

^C^ *term* corresponds to the T4 transcription terminator.

### VanU_G_ and VanR_G_ compete for binding to the *P*_*YG*_ resistance promoter

**Fig 6:**
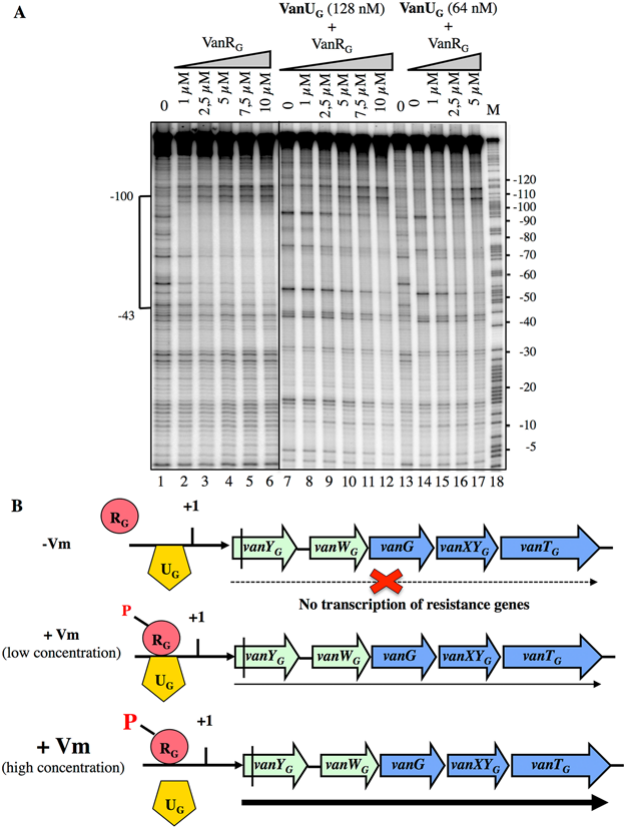
Competition between VanU_G_ and VanR_G_ for binding to the *P_YG_* resistance promoter. (A) DNase I footprinting analysis. A 233-bp DNA fragment was amplified from the *PYG* region using a labeled reverse primer (YG10) (S2 Table) to radiolabel the template strand. Increasing amounts of VanR_G_ and two fixed amounts of VanU_G_, indicated at the top, were incubated with the DNA probe. The bracket indicates the region protected from DNase I cleavage by VanU_G_ and/or VanR_G_ and the co-ordinates of protection relative to the transcriptional start site are indicated on the left. M is the A+G Maxam and Gilbert sequencing reaction lane of the probes used as a size marker and the nucleotide positions are indicated at the right. (B) Model for the binding competition between VanU_G_ and VanR_G_-P in the absence or in the presence of various concentrations of vancomycin (Vm).

Since VanU_G_ and VanR_G_ bound at overlapping sites of *P*_*YG*_, to assess a possible effect of VanR_G_ on the binding of VanU_G_, we performed DNaseI footprinting assays on the labeled *P*_*YG*_ probe with purified VanR_G_ and VanU_G_ (Fig 6). Low and medium concentrations (64 nM and 128 nM) of VanU_G_ which allow binding to *P*_*YG*_ were tested with increasing concentrations of VanR_G_. Upon addition of VanR_G_, the binding profile of VanU_G_ faded while that of VanR_G_ appeared and increased in a dose dependent manner (Fig 6A). In the reverse experiment two approriate concentrations of VanR_G_ were challenged by increasing concentrations of VanU_G_ and the binding of VanR_G_ decreased also in the presence of VanU_G_ (S4 Fig). In summary, VanU_G_ alone did not allow transcription of the resistance genes (Fig 6B). It thus appears that at a low concentration of vancomycin there was competition between VanU_G_ and VanR_G_, the latter being partially phosphorylated, transcription of *vanY*_*G*_ *W*_*G*_ *GXY*_*G*_ *T*_*G*_ was low. In contrast, at high concentrations of vancomycin, VanR_G_ was efficiently phosphorylated and able to displace VanU_G_ leading to maximal transcription of the resistance genes from the *P*_*YG*_ promoter.

### The presence of *vanU*_*G*_ reduces the fitness cost associated with expression of VanG-type resistance

To study the role of VanU_G_ in this sophisticated resistance mechanism, the fitness cost of BM4720(*ΔvanU*_*G*_) compared with that of BM4522 in monocultures in the absence and in the presence of vancomycin (1 μg/ml) was analysed by determination of the growth rates (Table 3). The results showed that the growth rates of both strains were indistinguishable in the absence of vancomycin indicating that non induced VanG-type resistance is not costly for the host. In contrast, in the presence of vancomycin, the relative growth rate of BM4720(*ΔvanU*_*G*_) (0.74) was significantly reduced when compared with that of BM4522 (0.93) indicating that increased expression of resistance was significantly more costly in the absence of *vanU*_*G*_.

**Table 3:** Growth rate.

	Growth rate ^a^		Relative growth rate^b^
Strain	NI	Vm1	
*E*. *faecalis* BM4522	0.027 ± 0.001	0.025 ± 0.001	0.926
*E*. *faecalis* BM4720 (*ΔvanU*_*G*_)	0.027 ± 0.001	0.020 ± 0.002	0.741

^a^ Exponential growth rate measured in the absence of antibiotic or in the presence of vancomycin (1μg/ml) (Vm1); average of at least four independent experiments ± standard deviations.

^b^ Relative growth rate was calculated as the ratio of the growth rate of the strain induced by 1μg/ml of vancomycin versus the non induced strain.

## Discussion

Among the ubiquitous two-component regulators, VanR/VanS-type systems are one of the rare to control expression of genes mediating antibiotic resistance [3]. In the VanG-type strains, a membrane associated sensor kinase (VanS_G_) which detects a signal associated with the presence of vancomycin in the environment and a cytoplasmic response regulator (VanR_G_) that acts as a transcriptional activator are also present (Fig 1) and functional (Fig 3) but there is, in addition, a VanU_G_ transcriptional regulator (Fig 1).

In the two main VanA- and VanB-type systems, the regulatory genes (*vanRS*) and the resistance genes are transcribed from independent and coordinately regulated promoters, but VanR is the only known direct regulator of the resistance genes [3, 8, 13]. In VanG-type strains, co-transcription of *vanURS*_*G*_ is repressed from *P*_*UG*_ by VanU_G_ (Fig 2 and Table 1) and expression of the resistance genes from *P*_*YG*_ is activated by VanR_G_ and repressed by VanU_G_ (Fig 5 and Table 2). Thus, VanU_G_ regulates the resistance genes both directly, by binding to the *P*_*YG*_ promoter region (Fig 4), and indirectly by repressing synthesis of VanR_G_S_G_ (Fig 5). Like other members of the XRE protein family (S3B Fig) [14–16], VanU_G_ binds to short repeated sequences which span the promoters (Fig 2A and 2B). Unlike the VanR and VanR_B_ proteins which bind to their own promoters [8, 13], VanR_G_ does not regulate its own expression (Fig 2). No sequences similar to the VanR_G_ consensus binding site are found in *P*_*UG*_ (Figs 2 and 4).

VanR_G_, as VanR and VanR_B_, belongs to the OmpR-PhoB subclass of response regulators that have the peculiarity to bind to their target promoters in the unphosphorylated or phosphorylated form [8, 13, 17, 18]. Phosphorylation of VanR and VanR_B_ enhances the affinity of the proteins for their respective regulatory *P*_*R*_ or *P*_*RB*_ and resistance *P*_*H*_ or *P*_*YB*_ promoter regions allowing increased transcription of the regulatory and resistance genes [8, 13]. In VanA-type strains, VanR and VanR-P bind to *P*_*R*_ and *P*_*H*_ regions which contain a single or two 12-bp conserved sites, respectively [13]. Comparison of the sequences of the *P*_*UG*_ and *P*_*YG*_ regions with the 12-bp consensus sequence spanned by VanR and VanR-P revealed three binding sites in the *P*_*YG*_ region with a consensus sequence (Fig 4B) similar to that in VanA-type resistance [13]. As for the regulatory *P*_*R*_ and resistance *P*_*H*_ promoters, the positioning of these sites in *P*_*YG*_ was upstream from the -35 motif. VanU_G_, VanR_G_, and VanR_G_-P protected overlapping regions, the two latter binding to *P*_*YG*_ a and b sites with a higher affinity than to the c site (Fig 4). There are only two sites in the *P*_*H*_ promoter but VanR generated a more extensive footprint (80 bp for *P*_*H*_) than VanR_G_ (42bp for *P*_*YG*_) likely due to higher cooperativity of VanR. Although not essential for binding in vitro, phosphorylation of VanR_G_ increased its affinity for the *P*_*YG*_ resistance promoter (Fig 4). In the *P*_*UG*_ promoter region no sequences similar to the consensus were found (Fig 2A) which could explain the absence of binding of VanR_G_ and low-level transcription from the regulatory promoter.

In many instances, regulation of gene transcription in *E*.*coli* occurs essentially through control of the phosphatase activity of the sensor [19, 20]. In VanA- and VanB-type strains, the level of phosphorylation of VanR and VanR_B_ is modulated by the kinase and phosphatase activities of the VanS and VanS_B_ sensors [7, 10, 21]. Phosphatase activity is critical for response regulators, such as VanR and VanR_B_, whose phosphorylated form is highly stable, to ensure that the protein is not permanently activated. In VanG-type strains, in the absence of VanU_G_, induction by vancomycin led to maximal VanT_G_ serine racemase (Fig 5) or CAT synthesis (Table 2) even at low concentrations of the inducer. Since in the absence of VanU_G_ there was no modulation of resistance genes transcription from the *P*_*YG*_ promoter, this suggests that a low amount of VanR_G_-P is sufficient to induce the resistance operon. VanU_G_ did not modulate VanR_G_ and VanS_G_ phosphorylation (Fig 4F) and was not phosphorylated by VanS_G_ (Fig 4E). Surprisingly, at least in vitro, the phosphatase activity of VanS_G_ was not very efficient (Fig 4D) in comparison with those of VanS or VanS_B_ [7, 9]. Expression of VanG-type resistance was thus inducible by vancomycin due to the presence of VanU_*G*_ as opposed to direct modulation of VanR activity by VanS in the other *van* operons. In the absence of vancomycin only VanU_G_ bound to the *P*_*YG*_ promoter; however when the concentration of vancomycin increased, VanR_G_ being more efficiently phosphorylated by VanS_G_, displaced progressively VanU_G_ allowing gradual transcription of the resistance genes (Fig 6) as it is likely the case with VanR'_G_, the VanR_G_ homolog encoded elsewhere in the chromosome. In *B*. *subtilis*, when both repressors SinR and SlrR are bound to the *degU* promoter, they can be displaced by the response regulator DegU leading to activation of the *degU* gene [22]. Also in *B*. *subtilis*, CcpC activates aconitase gene *citB* expression whereas CodY binds to its promoter and represses *citB* transcription [23]; PutR which is an activator essential for transcription of the *putBCP* operon for proline utilization is displaced by the CodY repressor [24].

VanU_G_ does not possess the characteristics of auxiliary regulators which can interact with histidine kinases, influencing signal perception and transduction. Nor does it interact with the response regulator to alter its phosphorylation status or its DNA binding ability, the recruitement of RNA polymerase on the promoter, or to sequester it through protein:protein interaction [25, 26]. The results presented here show that competition between the VanU_G_ repressor and the VanR_G_ activator for binding to the *P*_*YG*_ promoter may be responsible for the complex regulation of the resistance genes (Fig 6). This is an unusual example of rheostatic regulation of gene transcription due to binding competition between two regulators encoded in the same operon. It also elucidates an unsuspected strategy by which enterococcal clinical isolates regulate transcription of acquired genes for vancomycin resistance.

In previous work, we showed in VanB-type resistance that, despite the complex dual biochemical mechanism of resistance to vancomycin, its biological cost in enterococci is negligible when non induced, whereas a significant fitness reduction is observed when resistance is expressed in the presence of the inducer, the antibiotic itself [27]. Thus resistance is expressed exclusively when needed for bacterial survival. In VanG-type strains, tight regulation of resistance expression involves VanU_G_ which can thus be considered as a compensatory component, drastically reducing the biological cost associated with vancomycin resistance in the presence of antibiotic.

## Materials and Methods

### Bacterial strains, plasmids, and growth conditions

The origin and properties of the strains and plasmids are described in S1 Table. *Escherichia coli* TOP10 (Invitrogen, Groningen, The Netherlands) and NR698 (susceptible to vancomycin) [12] were used as a host for recombinant plasmids. *E*. *coli* BL21λDE3 [28], in which the T7 RNA polymerase gene is under the control of the inducible *lacUV5* promoter carries the pREP4 plasmid allowing co-expression of the GroESL chaperonin to optimize recombinant protein solubility [29]. *E*. *coli* TG1 RepA [30] was used as a host for constructions in the pAT944(pGhost9Ω*cat*) vector (S1 Table). Kanamycin (50μg/mL) was used as a selective agent for cloning PCR products in the pCR-Blunt vector (Invitrogen). Ampicillin was used to select pUC1813 [31]. pDR111 (gift from David Rudner, Harvard University), which harbors the *P*_*spank*_ promoter between two fragments of the *B*.*subtilis amyE* gene, is a derivative of the *P*_*spac-hy*_ plasmid pJQ43 containing an additional *lacO* binding site to achieve a better repression in the absence of the IPTG inducer. *P*_*spank*_ is a *lacI* repressible IPTG inducible-promoter for gene overexpression. Spectinomycin (60μg/mL) and chloramphenicol (10μg/mL) were added to the medium to prevent loss of plasmids derived from pDR111(*P*_*spank*_) and pAT944(*pGhost9Ωcat*), respectively. Enterococcus faecalis JH2-2 is a derivative of strain JH2 that is resistant to fusidic acid and rifampin [32]. In all experiments, strains were grown in brain heart infusion (BHI) at 37°C with shaking at 110 rpm.

### Promoter DNA labeling

Labeled *P*_*UG*_ (357 bp) and *P*_*YG*_ (233 bp) fragments were generated by PCR with BM4518 total DNA as a template and primer pairs VanG12-VanG126 and VanSG6-YG10 (S2 Table), respectively, using a combination of an unlabeled primer with an end-labeled primer (625nM) with T4 polynucleotide kinase (0.075 U/μl) (New England Biolabs) and [γ^32^P]-ATP (3000 Ci/mmol) (Perkin Elmer). The PCR reactions were carried out in a 50-μl volume and the products purified as described [8].

### Gel shift assay

Purified labeled PCR products corresponding to wild-type and mutated *P*_*UG*_ promoter region fragments were recovered from a 6% polyacrylamide gel and used as a probe for the gel shift assay after addition of 100 μl of ammonium acetate (0.5 M) diluted in Tris buffer (10 mM, pH8.5) overnight at 37°C. The *P*_*UG*_ and mutated *P*_*UG*_ probes (10,000cpm each) were incubated with various concentrations of purified VanU_G_ regulator at 30°C for 20min in 20 μl of 50mM Tris-HCl (pH7.8) containing 20 mM MgCl2 and 0.1 mM dithiothreitol (DTT). After addition of the DNA dye solution (40% glycerol, 0.025% bromophenol blue and 0.025 xylene cyanol), the mixture was loaded on a 7.5% polyacrylamide gel in the absence of protein denaturants. The gels were dried and analysed by autoradiography.

### DNase I footprinting

Complexes with the labeled promoter regions (5nM) were formed for 30 min at 30°C in 15 μl of buffer C (20 mM Hepes pH 8.0, 5 mM MgCl2, 50 mM potassium glutamate, 5 mM DTT, and 500μg/ml bovine serum albumin) using RNA polymerase of *E*. *coli* at 50 nM or VanU_G_, VanR_G_, or VanR_G_-P at increasing concentrations. For DNase I experiments, 1.5 μl of DNase I solution (1 μg ml^-1^ in 10 mM Tris-HCl, 10 mM MgCl2, 10 mM CaCl_2_, 125 mM KCl) were added and incubated at 30°C for 10s when the labeled promoter regions were alone, or for 20 s when when RNA polymerase or VanU_G_, VanR_G_ or VanR_G_-P were present in the mixture. The reaction was stopped and all the samples were extracted, precipitated, washed, resuspended, and loaded on a sequencing gel as described [8]. Protected bands were identified by comparing the migration with that of the same fragment treated for the A+G sequencing reaction [33]. The gels were analysed by autoradiography.

### Quantitative real-time RT-qPCR

Enterococci grown in 100 ml of brain heart infusion in 250-ml bottles, with and without vancomycin, at 37°C with shaking at 110 rpm to OD_600_ = 0.8 were harvested. RNA was prepared using the Fast RNA ProBlue kit (MBP Biomedicals) according to the manufacturer's protocol, treated with DNase (Turbo DNA-free, Invitrogen), and checked for the absence of contaminant DNA in a standard PCR, using the same primers as for the RT-PCR. RNA concentrations were determined by measuring absorbance with a NanoDrop2000 (ThermoScientific). cDNA synthesis and RT-qPCR were performed with a Light Cycler RNA amplification kit SYBR greenI (Roche Diagnostic GmbH) in a total reaction volume of 19μl with 0.5 μM gene-specific primers (VanG129-VanG102 for *vanU*_*G*_, VanRG2-VanRG10 for *vanR*_*G*_, VanSG2-VanSG10 for *vanS*_*G*_, and rpoB5-rpoB12 for *rpoB*) (S2 Table) according to the manufacturer's instructions. Amplification and detection of specific products were performed using the LightCycler sequence detection system (Roche) with the following cycle profile: 1cycle at 55°C for 20 min for the reverse transcription step, followed by 1 cycle at 95°C for 30 s, 45 cycles at 95°C for 5 s, 52°C for 15 s, and 72°C for 15 s. The level of every gene transcript was normalized relative to *rpoB* transcript levels.

### Overproduction and purification of VanU_G_, VanR_G_, and VanS_G_

Plasmids pAT940(pET28Ω*vanU*_*G*_), pAT941(pET28Ω*vanR*_*G*_), and pAT942(pET28Ω*vanS*_*G*_) (S1 Table) were introduced into *E*. *coli* BL21λDE3/pREP4 [29]. The transformants were grown in 1 liter of LB medium in Fernbach flasks with shaking at 110 rpm at 28°C until OD600 = 0.8, IPTG (1 mM) was added to induce protein production, and incubation was pursued for 4 h. *E*.*coli* crude protein extracts were loaded on 1-ml His-Trap fast-flow columns (GE, Healthcare) equilibrated with buffer A (50mM NaH_2_PO_4_ pH 7.5, 300 mM NaCl, 30 mM imidazole) and the proteins were eluted with an imidazole gradient (30mM-500mM). Fractions were dialysed against buffer B (50mM NaH_2_PO_4_ pH 7.5, 300 mM NaCl, 50% glycerol). Protein concentration was determined using the Bio-Rad protein assay [34].

### Autophosphorylation of VanS_G_

Autophosphorylation of VanS_G_ (40 μg) was performed in a final volume of 100 μl of buffer A (final concentrations: 50 mM Tris-HCl, 50mM KCl and 1 mM MgCl2, pH7.5). The reaction was initiated by the addition of 5 μl of ATP (1mM final) containing 200 μCi of [γ-32P]ATP and incubated at room temperature for 1 h. ATP was removed using 500 μl Sephadex G-50 spin column equilibrated with buffer A. The reaction was quenched by the addition of 5 μl of β-mercaptoethanol-stop solution (Sigma), followed by electrophoresis on 12% NuPAGE Novex Bis-Tris gels (Invitrogen) in MOPS buffer (1X), and autoradiography.

### Phosphorylation of VanU_G_ and VanR_G_ by VanS_G_

Phosphotransfer to purified VanU_G_ and VanR_G_ were carried out in buffer A. The reaction was initiated by the addition of 10 μl of the purified autophosphorylation reaction mixture of VanS_G_ (40 μg) described above to a 15 μl reaction mixture containing VanU_G_ or VanR_G_ (55 μg each). After incubation for various periods of times at room temperature, the phosphotransfer reactions were quenched by the addition of stop solution (Sigma) followed by electrophoresis on 12% NuPAGE Novex Bis-Tris gels (Invitrogen) in MOPS buffer (1X) and autoradiography.

### Phosphorylation of VanU_G_ and VanR_G_ by acetyl[^32^P]phosphate

VanU_G_ (220 μg) or VanR_G_ (225 μg) were incubated in 100 μl of buffer B (50 mM Tris-HCl, pH7.8, 20 mM MgCl2, 0.1 mM dithiothreitol) containing 178 pmol (3.3 μCi) of acetyl[^32^P]phosphate (Hartmann Analytical, Germany) at room temperature for 60 min. Excess acetyl[^32^P]phosphate was removed using Sephadex G-50 spin columns equilibrated with buffer B. Aliquots (10 μl) were withdrawn at designated time points, and the phosphorylation reactions were quenched with β-mercaptoethanol-stop solution followed by electrophoresis on 15% SDS-polyacrylamide gels and autoradiography.

### Hydrolysis of phospho-VanU_G_ and phospho-VanR_G_ by VanS_G_

The VanU_G_ (220 μg) and VanR_G_ (225 μg) response regulators were labelled with acetyl[^32^P]phosphate for 1 h at room temperature as described above, and 52 μg of VanS_G_ histidine kinase were added, and incubation was pursued for various periods of times. Aliquots (10 μl) were withdrawn at designated time points and the reactions were stopped, followed by electrophoresis on 15% SDS-polyacrylamide gels and autoradiography.

### Plasmid construction

The plasmids were constructed as follows.

#### Construction of pAT940, pAT941 and pAT942. pAT940(pET28Ω*vanU*_*G*_) and pAT941(pET28Ω*vanR*_*G*_)

A 225-bp *Bsa*I-*Xho*I fragment corresponding to the *vanU*_*G*_ coding sequence amplified with UG1 and UG2 (S2 Table) and a 705-bp *Bsa*I-*Xho*I fragment corresponding to the *vanR*_*G*_ coding sequence amplified by using oligonucleotides RG1 and RG2 (S2 Table) and BM4518 [11] total DNA as a template, were cloned in the *Nco*I and *Xho*I sites of modified pET28 [35] to generate plasmids pAT940(pET28Ω*vanU*_*G*_) and pAT941(pET28Ω*vanR*_*G*_). Oligodeoxynucleotide UG1 contained a *Bsa*I restriction site designed to generate a cohesive end compatible with *Nco*I and 16 bases complementary to codons 1–6 of *vanU*_*G*_ of BM4518 (S2 Table). Oligodeoxynucleotide UG2 contained a *Xho*I site replacing the TGA stop codon and 21 bases complementary to codons 69–75 of *vanU*_*G*_. Oligodeoxynucleotide RG1 contained a *Bsa*I restriction site designed to generate a cohesive end compatible with *Nco*I and 16 bases complementary to codons 1–6 of *vanR*_*G*_ of BM4518. Oligodeoxynucleotide RG2 contained a *Xho*I site replacing the TGA stop codon and 21 bases complementary to codons 229–235 of *vanR*_*G*_.

#### pAT942(pET28Ω*vanS*_*G*_)

A cytoplasmic portion of the *vanS*_*G*_ gene of strain BM4518 was amplified using BM4518 total DNA as a template and primer pair SG1-SG3 (S2 Table). Oligodeoxynucleotide SG1 contained a *Bsa*I restriction site designed to generate a cohesive end compatible with *Nco*I, and 16 bases complementary to codons 88–93 of *vanS*_*G*_. Oligodeoxynucleotide SG3 contained a *Xho*I site in place of the TAG stop codon and 21 bases complementary to codons 361–367 of *vanS*_*G*_. The 842-bp pCR product from *vanS*_*G*_ was digested by *Bsa*I and *Xho*I and cloned between the *Nco*I and *Xho*I restriction sites of plasmid pET28 to generate plasmid pAT942(pET28Ω*vanS*_*G*_).

#### Construction of pAT944(pGhost9Ω*cat*)

The XbaI cassette containing the chloramphenicol acetyltransferase *cat* gene with its own promoter was amplified from DNA of plasmid pAT943(pUC1318Ω*Pcat*) with primers pG9CAT_NH2_ and pG9CAT_COOH_ (S2 Table) which contain a XbaI restriction site allowing the replacement of the XbaI fragment containing the erythromycin resistance gene in pGhost9 [36] to generate plasmid pAT944(pGhost9Ω*cat* **)**.

#### Construction of pAT945(pGhost9CmΩ*ΔvanU*_*G*_), pAT946(pGhost9CmΩΔ*vanR*_*G*_), pAT947(pGhost9CmΩΔ*vanS*_*G*,_), and pAT973(pGhost9CmΩΔ*vanR'*_*G*_)

The *vanU*_*G*_, *vanR*_*G*_, and *vanS*_*G*_ genes of the *vanG* operon and *vanR'*_*G*_ from BM4518 were inactivated by deletion using splicing-by-overlap extension PCR in two steps and cloned into the thermosensitive shuttle plasmid pAT944(pGhost9Ω*cat*) using XhoI and PstI restriction sites to generate plasmids pAT945(pGhost9CmΩ**Δ** *vanU*_*G*_), pAT946(pGhost9CmΩ**Δ** *vanR*_*G*_), pAT947(pGhost9CmΩ**Δ** *vanS*_*G*_), and pAT973(pGhost9CmΩ**Δ** *vanR'*_*G*_). The primers used for the construction of the deletant alleles and the extent of the deletions are reported in S2 Table. A SmaI restriction site was added in the primers to screen for integration in the corresponding chromosomal gene. Briefly, the remnants of the *vanU*_*G*_, *vanR*_*G*_, *vanS*_*G*_ and *vanR'*_*G*_ genes of BM4518 were first amplified from total DNA of BM4518 as a template using primers UG3-UG4 and UG5-UG6 for **Δ** *vanU*_*G*_, UG3-RG4 and RG5-RG7 for **Δ** *vanR*_*G*_, SG4-SG5 and SG6-SG7 for **Δ** *vanS*_*G*_, RG10-RG11 and RG12-RG13 for **Δ** *vanR'*_*G*_ and, in a second step, the resulting PCR products were amplified with UG3 plus UG6, UG3 plus RG7, SG4 plus SG7, and RG10 plus RG13 respectively, to obtain **Δ** *vanU*_*G*_, **Δ** *vanR*_*G*_, **Δ** *vanS*_*G*_ and **Δ** *vanR'*_*G*_.

#### Construction of pAT949 and derivatives

Plasmid pAT949(pDR111Ω*P*_*spank*_ *cat*) was constructed by cloning the HindIII-SphI fragment of pAT948(pUC1813Ω*cat*) carrying the *cat* cassette in pDR111(*P*_*spank*_) digested with the same enzymes allowing a directional cloning of the *cat* reporter gene under the control of the inducible *P*_*spank*_ promoter.

#### pAT950 (pDR111Ω*P*_*spank*_ *termcat*)

A 66-bp HindIII-SalI fragment corresponding to the transcription terminator of gene 32 from bacteriophage T4 [37] was amplified by PCR with oligodeoxynucleotides T4F-HindIII and T4R-SalI/NheI (S2 Table). Primer T4F-HindIII contained HindIII and NheI restriction sites. Primer T4R-SalI/NheI contained SalI and NheI restriction sites. The HindIII and SalI restriction sites allowed directional cloning of the transcription terminator (term) from bacteriophage T4 under the control of the inducible *P*_*spank*_ promoter and upstream from the *cat* reporter gene of the pAT949(pDR111Ω*P*_*spank*_ *cat*) shuttle vector.

#### pAT951(pDR111Ω*P*_*spank*_ *vanU*_*G*_ *cat*)

The *vanU*_*G*_ gene of BM4518 was amplified using primer pair UG_NH2_ and UG_COOH_ (S2 Table) and total DNA of the corresponding strain as a template. Oligodeoxynucleotide UG_NH2_ contained BsaI and HindIII restriction sites, a RBS, and 6 bases complementary to *vanU*_*G*_ including the ATG (translation initiation) codon. Oligodeoxynucleotide UG_COOH_ harbored SalI and NheI restriction sites, the stop codon, and 15 bases complementary to the 3’ end sequence of *vanU*_*G*_ from BM4518. The BsaI and SalI restriction sites allowed directional cloning of a 249-bp fragment of *vanU*_*G*_ downstream from the inducible *P*_*spank*_ promoter and upstream from the *cat* gene of the pAT949(pDR111Ω*P*_*spank*_ *cat*) shuttle vector to generate pAT951(pDR111Ω*P*_*spank*_ *vanU*_*G*_ *cat*).

#### pAT952(pDR111Ω*P*_*spank*_ *termP*_*UG*_ *cat*) and pAT953(pDR111Ω*P*_*spank*_ *vanU*_*G*_ *P*_*UG*_ *cat*)

The regulatory *P*_*UG*_ (183 bp) promoter was amplified by PCR from BM4518 total DNA with oligodeoxynucleotides PUG1 and PUG2 (S2 Table). Primers PUG1 and PUG2 contained a NheI and a SalI restriction site, respectively, which allowed directional cloning of *P*_*UG*_ upstream from the *cat* gene of pAT950(pDR111Ω*P*_*spank*_ *termcat*) to generate pAT952(pDR111Ω*P*_*spank*_ *termP*_*UG*_ *cat*) or allowed directional cloning of *P*_*UG*_ downstream from *vanU*_*G*_ and upstream from the *cat* reporter gene of pAT951(pDR111Ω*P*_*spank*_ *vanU*_*G*_ *cat*) to generate pAT953(pDR111Ω*P*_*spank*_ *vanU*_*G*_ *P*_*UG*_ *cat*).

#### pAT954(pDR111Ω*P*_*spank*_ *vanR*_*G*_ *P*_*UG*_ *cat*)

A 754-bp HindIII-NheI fragment corresponding to the *vanR*_*G*_ coding sequence with its RBS, initiation and stop codons was amplified by PCR from BM4518 with oligodeoxynucleotides RG_NH2_ and RG_COOH_ (S2 Table). Primer RG_NH2_ contained a HindIII restriction site. Primer RG_COOH_ comprised SalI and NheI restriction sites, the stop codon, and 14 bases complementary to the 3’ end of *vanR*_*G*_ from BM4518. The HindIII and NheI restriction sites allowed directional cloning of the *vanR*_*G*_ gene under the control of the inducible *P*_*spank*_ promoter and upstream from *P*_*UG*_ and the *cat* gene of pAT952(pDR111Ω*P*_*spank*_ *termP*_*UG*_ *cat*).

#### pAT956(pDR111Ω*P*_*spank*_ *vanUR*_*G*_ *P*_*UG*_ *cat*), pAT958(pDR111Ω*P*_*spank*_ *vanRS*_*G*_ *P*_*UG*_ *cat*), pAT960(pDR111Ω*P*_*spank*_ *vanURS*_*G*_ *P*_*UG*_ *cat*) pAT961(pDR111Ω*P*_*spank*_ *vanRS*_*G*_ *P*_*YG*_ *cat*)and pAT962(pDR111Ω*P*_*spank*_ *vanURS*_*G*_ *P*_*YG*_ *cat*)

The *vanUR*_*G*_, *vanRS*_*G*_, and *vanURS*_*G*_ genes of BM4518 were amplified using primer pairs UG_NH2_-RG_COOH_, RG_NH2_-SG_COOH_, and UG_NH2_-SG_COOH_ (S2 Table), respectively, and BM4518 total DNA as a template. Oligodeoxynucleotides UG_NH2_ and RG_NH2_ harbored a HindIII restriction site and 21 bases complementary to the sequence upstream from *vanU*_*G*_ or 17 bases complementary to the sequence upstream from *vanR*_*G*_. Primers RG_COOH_ and SG_COOH_ contained each SalI and NheI restriction sites, the stop codon and 14 or 13 bases complementary to the 3' end of respectively *vanR*_*G*_ and *vanS*_*G*_ of BM4518. The HindIII and SalI restriction sites allowed directional cloning of *vanUR*_*G*_, *vanRS*_*G*_, and *vanURS*_*G*_ upstream from the *cat* reporter gene of shuttle vector pAT949(pDR111Ω*P*_*spank*_ *cat*) carrying the inducible *P*_*spank*_ promoter to generate pAT955(pDR111Ω*P*_*spank*_ *vanUR*_*G*_ *cat*), pAT957(pDR111Ω*P*_*spank*_ *vanRS*_*G*_ *cat*), and pAT959(pDR111Ω*P*_*spank*_ *vanURS*_*G*_ *cat*). The 183-bp NheI-SalI fragment carrying the *P*_*UG*_ promoter obtained above by amplification was cloned in pAT955(pDR111Ω*P*_*spank*_ *vanUR*_*G*_ *cat*), pAT957(pDR111Ω*P*_*spank*_ *vanRS*_*G*_ *cat*), and pAT959(pDR111Ω*P*_*spank*_ *vanURS*_*G*_ *cat*) digested with the same enzymes to generate pAT956(pDR111Ω*P*_*spank*_ *vanUR*_*G*_ *P*_*UG*_ *cat*), pAT958(pDR111Ω*P*_*spank*_ *vanRS*_*G*_ *P*_*UG*_ *cat*), and pAT960(pDR111Ω*P*_*spank*_ *vanURS*_*G*_ *P*_*UG*_ *cat*). The 177-bp NheI-SalI fragment carrying the *P*_*YG*_ resistance promoter amplified by PCR from BM4518 DNA with primers PYG1 and PYG2 (S2 Table) was cloned in pAT957(pDR111Ω*P*_*spank*_ *vanRS*_*G*_ *cat*), and pAT959(pDR111Ω*P*_*spank*_ *vanURS*_*G*_ *cat*) digested with the same enzymes to generate, respectively, pAT961(pDR111Ω*P*_*spank*_ *vanRS*_*G*_ *P*_*YG*_ *cat*)and pAT962(pDR111Ω*P*_*spank*_ *vanURS*_*G*_ *P*_*YG*_ *cat*).

#### pAT964(pDR111Ω*P*_*spank*_ *vanU*_*G*_ *termcat*), pAT965(pDR111Ω*P*_*spank*_ *vanU*_*G*_ *termP*_*UG*_ *cat*), pAT966(pDR111Ω*P*_*spank*_ *vanR*_*G*_ *termP*_*UG*_ *cat*), pAT967(pDR111Ω*P*_*spank*_ *vanUR*_*G*_ *termP*_*UG*_ *cat*), pAT968(pDR111Ω*P*_*spank*_ *vanURS*_*G*_ *termP*_*UG*_ *cat*), pAT969(pDR111Ω*P*_*spank*_ *vanRS*_*G*_ *termP*_*UG*_ *cat*), pAT970(pDR111Ω*P*_*spank*_ *vanURS*_*G*_ *termP*_*YG*_ *cat*), and pAT971 (pDR111Ω*P*_*spank*_ *vanRS*_*G*_ *termP*_*YG*_ *cat*)

The NheI terminator fragment amplified by PCR with oligodeoxynucleotides T4F-NheI and T4R-NheI/KpnI (S2 Table) was cloned, respectively, in pAT951(pDR111Ω*P*_*spank*_ *vanU*_*G*_ *cat*), pAT953(pDR111Ω*P*_*spank*_ *vanU*_*G*_ *P*_*UG*_ *cat*), pAT954(pDR111Ω*P*_*spank*_ *vanR*_*G*_ *P*_*UG*_ *cat*), pAT956(pDR111Ω*P*_*spank*_ *vanUR*_*G*_ *P*_*UG*_ *cat*), pAT960(pDR111Ω*P*_*spank*_ *vanURS*_*G*_ *P*_*UG*_ *cat*), pAT958(pDR111Ω*P*_*spank*_ *vanRS*_*G*_ *P*_*UG*_ *cat*), pAT962(pDR111Ω*P*_*spank*_ *vanURS*_*G*_ *P*_*YG*_ *cat*) and pAT961(pDR111Ω*P*_*spank*_ *vanRS*_*G*_ *P*_*YG*_ *cat*) digested with NheI.

#### pAT972(pDR111Ω*P*_*spank*_ *vanUS*_*G*_ *termcat*)

The 1,144-bp fragment containing the *vanS*_*G*_ gene of BM4518 was amplified using primer pair SG_NH2_-SG_COOH_ (S2 Table) and total DNA of the corresponding strain as a template. The NheI and SalI restriction sites allowed directional cloning of *vanS*_*G*_ downstream from the *vanU*_*G*_ gene and upstream from the *cat* gene of pAT951(pDR111Ω*P*_*spank*_ *vanU*_*G*_ *cat*) to generate pAT963(pDR111Ω*P*_*spank*_ *vanUS*_*G*_ *cat*).

The EcoRI fragment harboring the *vanUS*_*G*_ *'* genes from pAT963(pDR111Ω*P*_*spank*_ *vanUS*_*G*_ *cat*) was replaced by the EcoRI fragment carrying the *vanRS*_*G*_ *'* genes of pAT971(pDR111Ω*P*_*spank*_ *vanRS*_*G*_ *termP*_*YG*_ *cat*) to generate pAT972(pDR111Ω*P*_*spank*_ *vanUS*_*G*_ *termcat*).

### Construction of strains

Plasmids pDR111, pAT949, pAT950, pAT952, pAT964, pAT965, pAT966, pAT967, pAT968, pAT969, pAT970, pAT971, and pAT972 were introduced by transformation into vancomycin susceptible *E*. *coli* NR698 and transformants were selected on agar containing chloramphenicol (10 g/ml) or ampicillin (100 μg/ml, for pDR111) (Tables 1 and 2).

In Gram-positive bacteria, pGhost9 [36] which replicates at 28°C but is lost above 37°C, allowed construction of *E*.*faecalis* BM4522 derivatives by insertional inactivation. Plasmids pAT945(pGhost9CmΩΔ*vanU*_*G*_), pAT946(pGhost9CmΩΔ*vanR*_*G*_), and pAT947(pGhost9CmΩΔ*vanS*_*G*_) were electrotransformed into *E*. *faecalis* BM4522 [11] to generate, respectively, BM4720(Δ*vanU*_*G*_), BM4721(Δ*vanR*_*G*_), and BM4722(Δ*vanS*_*G*_) (S1 Table). Plasmid pAT973(pGhost9CmΩΔ*vanR'*_*G*_) was electrotransformed into *E*. *faecalis* BM4721(Δ*vanR*_*G*_) to generate the double mutant BM4723(Δ*vanR*_*G*_, Δ*vanR'*_*G*_). Transformants were selected at the permissive temperature (28°C) on M17 plates containing 10g/ml of chloramphenicol and 0.5% glucose. A colony of each transformant was inoculated into 50 ml of M17 broth containing 0.5% glucose and incubated for 2h at 28°C. The culture was then shifted to a non-permissive temperature (42°C) for 2 h and integrants, following a first recombination event, were selected at 42°C on M17 agar containing chloramphenicol (10g/ml). Plasmid excision, by a second recombination event, was favored by subculturing at 28°C in the absence of chloramphenicol and plasmid loss was screened for by plating at 42°C on M17-glucose followed by replica plating on chloramphenicol. The integration locus was determined by PCR following digestion with SmaI and sequencing.

### Enzyme assays

For preparation of extracts, 8 ml of an overnight culture were added to 100 ml of broth in the absence or in the presence of vancomycin and strains were grown until OD_600_ = 0.8 in 250 ml bottles with shaking at 110 rpm. The cells were harvested by centrifugation, washed in 0.1M phosphate buffer pH 7.0, resuspended in the same buffer, lysed by sonication, followed by centrifugation at 10,000 g during 45 min. The resuspended pellet for VanT_G_ racemase [11] and supernatant for CAT activity, were assayed as described [38].

### Genome sequencing, assemblies and annotation

Total DNA from BM4518 and BM4522 strains was purified and sequencing library preparation was carried out using the Nextera DNA Sample Preparation kit (Illumina, San Diego, CA), according to manufacturer’s specifications. Quality and quantity of each sample library was measured on an Agilent Technologies 2100 Bioanalyzer (Santa Clara, CA). Libraries were normalized to 2nM, multiplexed and subjected to 250-bp paired end sequencing (Illumina MiSeq). On average, 5 million high-quality paired-end reads were collected for each strain, representing >220-fold coverage of the ~2.9 Mb genomes. Reads were assembled *de novo* utilizing CLC Genomics Workbench (CLC bio, Cambridge, MA). Functional annotations were performed using a custom pipeline as described previously [39].

### Determination of growth rates

Growth rates were determined in microplates coupled to a spectrophotometer iEMS reader (Labsystems). Strains were grown overnight at 37°C without or with 1 μg/ml of vancomycin. The cultures were diluted at OD 0.15 into 10 ml of broth without or with vancomycin (1μg/ml) and grown at 37°C with shaking until the beginning of the stationary phase. The cultures were diluted 1/1,000 to inoculate 10^5^ bacteria into 200 μl of broth in a 96-well microplate that was incubated overnight at 37°C with shaking. Absorbance was measured at 600 nm every 3 min. Each culture was replicated three times in the same microplate. Growth rates performed in three independent experiments were determined at the beginning of the exponential phase and the relative growth rates were calculated as the ratio of the growth rate of the strain induced by vancomycin versus that of the non induced strain.

## Supporting Information

S1 Fig Effect of mutations in the *PUG* promoter regulatory region on the in vitro binding of VanU_G_. (A) Sequence of the wild-type (WT) and mutated promoter regions. The two 14-bp imperfect inverted repeats corresponding to the putative binding sites are indicated in orange and pink and by arrows. A DNA fragment (197 bp) was obtained with PUG3 plus labeled VanG126 and mutated PUG5 plus labeled VanG126 primers (S2 Table) leading to the WT and corresponding mutated (mutant 1) promoter region, respectively. A DNA fragment (293 bp) was obtained with labeled VanG12 plus PUG4 and labeled VanG12 plus mutated PUG6 primers (S2Table) leading to the WT and corresponding mutated (mutant 2) promoter region, respectively. Numbering relative to the transcription start site is indicated above the sequences. Only bases differing from the WT sequence are shown in the mutated fragments. (B) Gel shift analysis. The labeled fragments corresponding to the WT and mutated (mutant 1 and mutant 2) promoter regions were incubated in the absence or in the presence of decreasing concentrations of purified VanU_G_ protein indicated above the lanes.
(TIF)

S2 Fig Binding of δ70 RNA polymerase of *E*. *coli* to (A) the *PUG* regulatory and (B) *PYG* resistance promoters by DNase I footprinting analysis. (A) A 357-bp DNA fragment was amplified from the *P*_*UG*_ promoter region using a labeled reverse primer (VanG126) (S2 Table) to radiolabel the template strand and the DNA probe was incubated without and with δ70 RNA polymerase at 50 nM. (B) A 233-bp DNA fragment was amplified from the *P*_*YG*_ promoter region using a labeled reverse primer (YG10) (S2 Table) to radiolabel the template strand and the DNA probe was analysed similarly. The brackets indicate the regions protected from DNase I cleavage by δ70 RNA polymerase, and the co-ordinates of protection relative to the transcriptional start site are indicated on the right. M is the A+G Maxam and Gilbert sequencing reaction lane of the probes used as a size marker and the nucleotide positions are indicated at the left. RNAP, RNA polymerase.
(TIF)

S3 Fig Comparison of the deduced amino acid sequences of VanR_G_ with VanR'_G_ (A) and of VanU_G_ from *E*. *faecalis* BM4518 with Cro/CI*cd* from *Clostridium difficile* (77% identity, GenBank N° EQJ96019) and Cro/CI*bf* from *Butyvibrio fibrisolvens* (52% identity, GenBank N° WP_022757627) (B). Identical amino acids are indicated by dashes below the alignment.
(DOC)

S4 Fig Competition between VanR_G_ and VanU_G_ for binding to the *PYG* resistance promoter by DNase I footprinting. A 233-bp DNA fragment was amplified from the *P*_*YG*_ region using a labeled reverse primer (YG10) (S2 Table) to radiolabel the template strand. Increasing amounts of VanU_G_ and two fixed amounts of VanR_G_ indicated at the top were incubated with the DNA probe. The bracket indicates the region protected from DNase I cleavage by VanR_G_ and/or VanU_G_ and the co-ordinates of protection relative to the transcriptional start site are indicated on the left. M is the A+G Maxam and Gilbert sequencing reaction lane of the probes used as a size marker and the nucleotide positions are indicated at the right.
(TIF)

S1 Table Bacterial strains and plasmids. (DOC)

S2 Table Oligonucleotide primers used. (DOC)
