# Supplementary figures and images for: Competition between VanUG Repressor and VanRG Activator Leads to Rheostatic Control of vanG Vancomycin Resistance Operon Expression
Source: PLoS Genet. 2015 Apr 21;11(4):e1005170. doi: 10.1371/journal.pgen.1005170 (PMC4405338; doi:10.1371/journal.pgen.1005170)

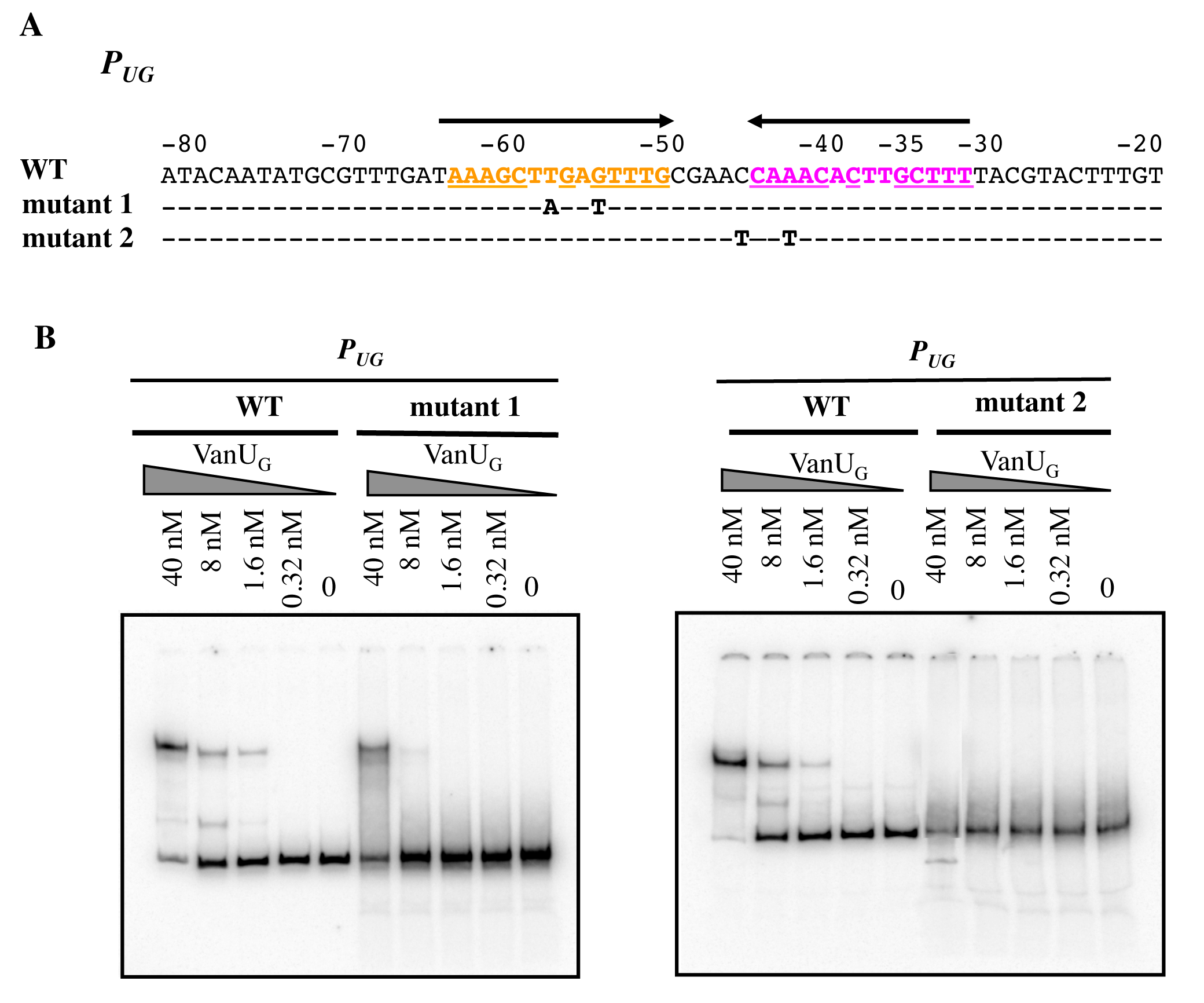

Supplement: S1 Fig — (A) Sequence of the wild-type (WT) and mutated promoter regions. The two 14-bp imperfect inverted repeats corresponding to the putative binding sites are indicated in orange and pink and by arrows. A DNA fragment (197 bp) was obtained with PUG3 plus labeled VanG126 and mutated PUG5 plus labeled VanG126 primers (S2 Table) leading to the WT and corresponding mutated (mutant 1) promoter region, respectively. A DNA fragment (293 bp) was obtained with labeled VanG12 plus PUG4 and labeled VanG12 plus mutated PUG6 primers (S2Table) leading to the WT and corresponding mutated (mutant 2) promoter region, respectively. Numbering relative to the transcription start site is indicated above the sequences. Only bases differing from the WT sequence are shown in the mutated fragments. (B) Gel shift analysis. The labeled fragments corresponding to the WT and mutated (mutant 1 and mutant 2) promoter regions were incubated in the absence or in the presence of decreasing concentrations of purified VanUG protein indicated above the lanes. (TIF) [file pgen.1005170.s001.tif]

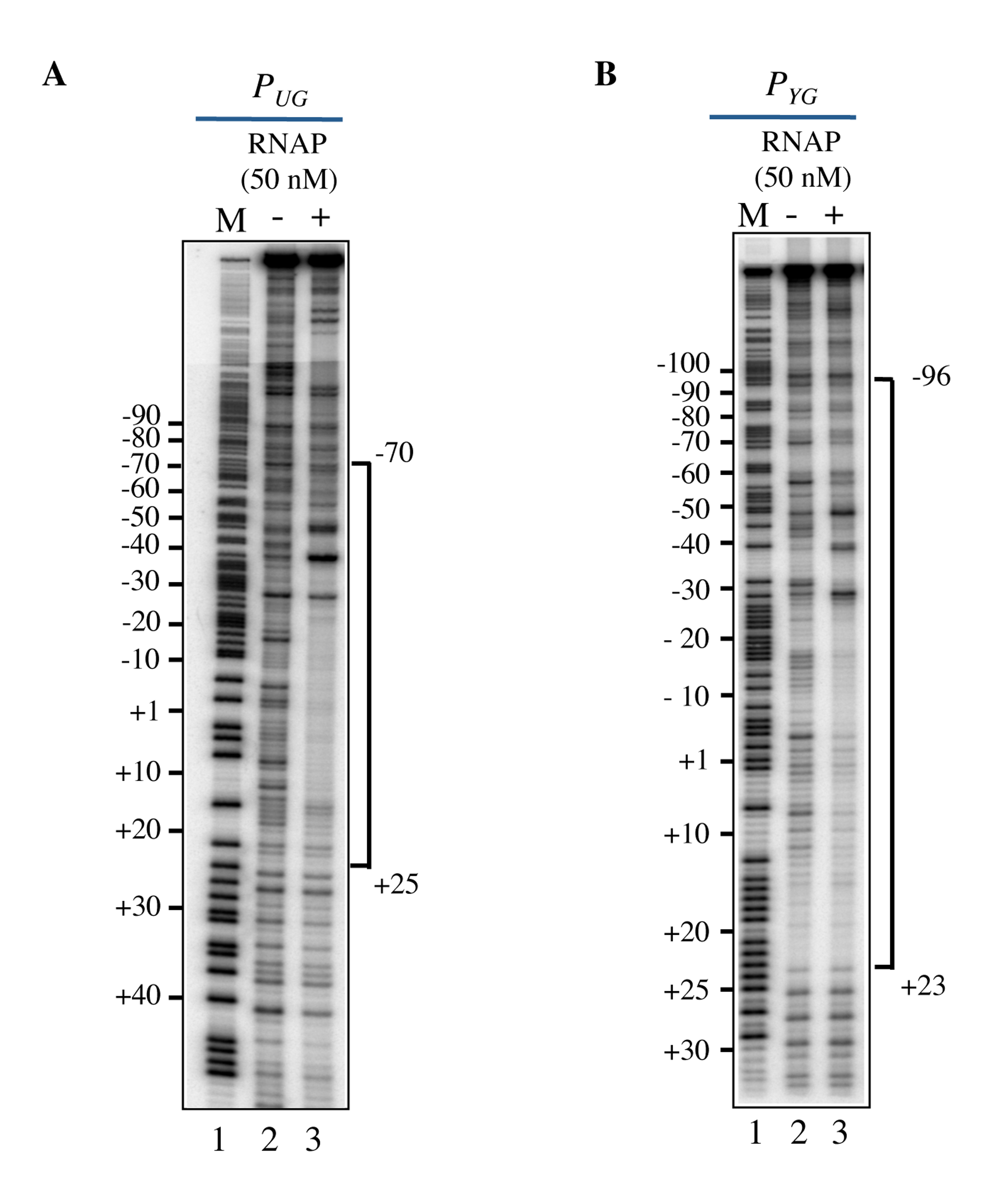

Supplement: S2 Fig — (A) A 357-bp DNA fragment was amplified from the P UG promoter region using a labeled reverse primer (VanG126) (S2 Table) to radiolabel the template strand and the DNA probe was incubated without and with δ70 RNA polymerase at 50 nM. (B) A 233-bp DNA fragment was amplified from the P YG promoter region using a labeled reverse primer (YG10) (S2 Table) to radiolabel the template strand and the DNA probe was analysed similarly. The brackets indicate the regions protected from DNase I cleavage by δ70 RNA polymerase, and the co-ordinates of protection relative to the transcriptional start site are indicated on the right. M is the A+G Maxam and Gilbert sequencing reaction lane of the probes used as a size marker and the nucleotide positions are indicated at the left. RNAP, RNA polymerase. (TIF) [file pgen.1005170.s002.tif]

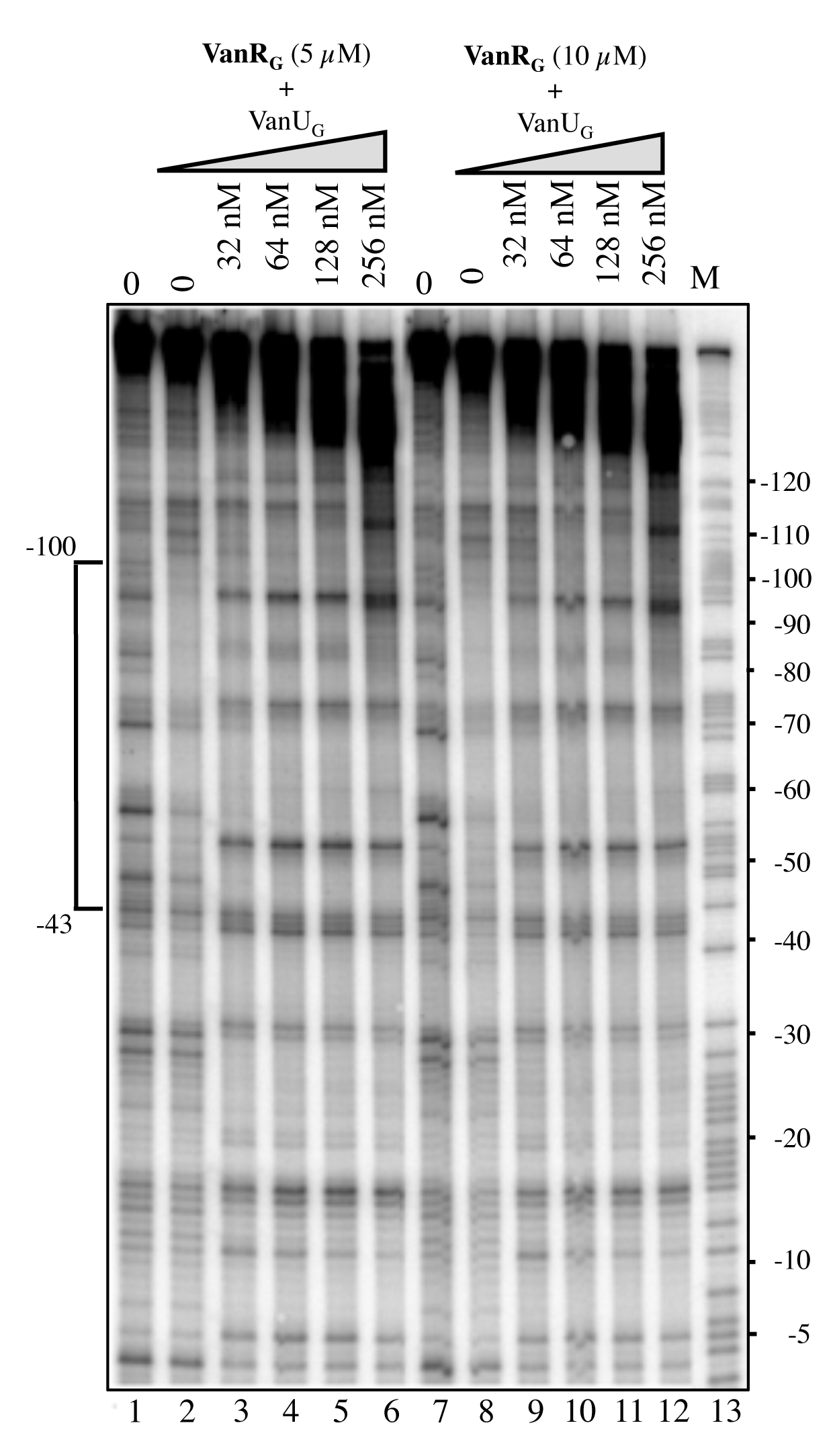

Supplement: S4 Fig — A 233-bp DNA fragment was amplified from the P YG region using a labeled reverse primer (YG10) (S2 Table) to radiolabel the template strand. Increasing amounts of VanUG and two fixed amounts of VanRG indicated at the top were incubated with the DNA probe. The bracket indicates the region protected from DNase I cleavage by VanRG and/or VanUG and the co-ordinates of protection relative to the transcriptional start site are indicated on the left. M is the A+G Maxam and Gilbert sequencing reaction lane of the probes used as a size marker and the nucleotide positions are indicated at the right. (TIF) [file pgen.1005170.s004.tif]
