# Supplementary material for: Competition between VanUG Repressor and VanRG Activator Leads to Rheostatic Control of vanG Vancomycin Resistance Operon Expression
Source: PLoS Genet. 2015 Apr 21;11(4):e1005170. doi: 10.1371/journal.pgen.1005170 (PMC4405338; doi:10.1371/journal.pgen.1005170)
Supplement: S3 Fig — Identical amino acids are indicated by dashes below the alignment. (DOC) [file pgen.1005170.s003.doc]

**A**

VanR_G_ MNEKILIVDDEKEIADLIELYLKNDGYKVYKFYNGIDALKCVESEKMDLAILDVMLPDVDGFHICQKIRERYFYP

VanR'_G_ MAERILLVDDEQEIADLLEVYLTSDGYEVEKFYRGKPALESMANNQFDLAVLDVMLPDIDGFQMVKKAREKYFFP

- - -- ---- ----- - -- --- - --- - -- --- ------- --- - -- -- -

VanR_G_ IIMLTAKVEDADKIMGLTIGADDYITKPFNPLEVAARVKTQLRRYVCYNNAADIEKENVLVTEYDINGLVINKNT

VanR'_G_ IILLTAKIEDIDKITGLTLGADDYITKPFNPLEVVARIKTQLRRFRKYNSQASL=QQETEGTEIDIHGLVINKIH

-- --- ---- -- --- --------------- -- ------ -- - -- -- ------

VanR_G_ HKCTLYGKAVTLTPIEFSVLWYLCENRGKVISSEELFENVWGEKFLDNN=NTVMAHIGRLREKLKEPARNPKFIK

VanR'_G_ HTSKLYGEDIFLTPLEFSILWYLAERQGEVVPAEILFEEIWGEKYLENNGNTVMAHIGRLREKLKEPPRKPKFIK

-- --- --- --- ---- - - - - --- ---- - -- ----------------- - -----

VanR_G_ TVWGVGYTIEE

VanR'_G_ TVWGVGYKID

-------

**B**

VanU_G_ MRVSYNKLWKLLIDRDMKKGELREAVGVSKSTFAKLGKNENVSLTVLLAICEYLNCDFGDIIEALPETPDKERDS

Cro/CI*cd* MRVSYNKLWKMLIDRGMKKSQLREAVGASKSTFAKLGKNENVTLPVLLDICEYLQCDFGDIMEALPDEVNKECED

Cro/CI*bf* MKVSYNKLWKLLIDMNLNKTKLREMAQMSPNTMAKLGKNETISMDIILRICEVLKCDVGDIMETIADEEDSNT

- -------- --- - --- - - ------- - --- - -- --- -

S3 Fig.
