# Supplementary material for: Competition between VanUG Repressor and VanRG Activator Leads to Rheostatic Control of vanG Vancomycin Resistance Operon Expression
Source: PLoS Genet. 2015 Apr 21;11(4):e1005170. doi: 10.1371/journal.pgen.1005170 (PMC4405338; doi:10.1371/journal.pgen.1005170)
Supplement: S1 Table — (DOC) [file pgen.1005170.s005.doc]

**Table S1. Bacterial strains and plasmids**

| Strain or plasmid | Relevant properties | Reference |
| --- | --- | --- |
|  |  | or source |
| Strains |  |  |
| *E. coli* |  |  |
| TOP10 | F^-^ *mcr*A ∆(*mrr-hsd*RMS-*mcr*BC) φ80**∆M15 ∆*lac*X74 *deo*R *rec*A1 *ara*D139  ∆(*ara-leu*)7697 *gal*U *gal*K *rps*L *end*A1 *nup*G | Invitrogen |
| NR698 | MC4100 *imp4213*, Vm^S^ | [12] |
| BL21λDE3 | F- *omp*T *hsd*S_B_(r_B_^-^m_B_^-^) *gal dcm* (DE3) | [28] |
| BL21λDE3/pREP4 | F- *omp*T *hsd*S_B_(r_B_^-^m_B_^-^) *gal dcm* (DE3) pREP4 (Km^R^) | [29] |
| TG1 RepA | *supE hsdD5 thi* (∆*lac-proAB*) F' (*traD36 proAB-lacZ∆M15*) | [30] |
| *E. faecalis* |  |  |
| JH2-2 | Fus^R^ Rif^R^ | [32] |
| BM4518 | Vm^R^ Te^S^ (VanG-type) | [11] |
| BM4522 | BM4518 X JH2-2 Vm^R^ Te^S^ (VanG-type) | [11] |
| BM4720 | BM4522∆*vanU_G_* (in frame deletion) | This study |
| BM4721 | BM4522∆*vanR_G_* (in frame deletion) | This study |
| BM4722 | BM4522∆*vanS_G_* (in frame deletion) | This study |
| BM4723 | BM4522∆*vanR_G_* ∆*vanR'_G_* (in frame deletion) | This study |
|  |  |  |
| Plasmids |  |  |
| pCR-Blunt | Km^R^, Zeocin^R^, *ori*R from ColE1, *lac*Zα, *ccd*B | Invitrogen |
| pET28 | Vector for overexpressing His-tagged proteins using a T7 bacteriophage promoter, Ap^R^ | [35] |
| pGhost9 | Em^R^, *ori*TS | [36] |
| pAT940 | 225-bp NcoI-XhoI PCR fragment (*vanU_G_*) of BM4518 cloned in pET28 | This study |
| pAT941 | 705-bp NcoI-XhoI PCR fragment (*vanR_G_)* of BM4518 cloned in pET28 | This study |
| pAT942 | 842-bp NcoI-XhoI PCR fragment (*vanS_G_)* of BM4518 cloned in pET28 | This study |
| pAT943 | 1660-bp ClaI fragment of *cat* from pC194 in pUC1318 | This study |
| pAT944 | 986-bp XbaI PCR fragment (*cat*) of pAT943 in pGhost9, Cm^R^ | This study |
| pAT945 | 166-bp XhoI-PstI PCR fragment of truncated *vanU_G_* cloned in pAT944 | This study |
| pAT946 | 613-bp XhoI-PstI PCR fragment of truncated *vanR_G_* cloned in pAT944 | This study |
| pAT947 | 1030-bp XhoI-PstI PCR fragment of truncated *vanS_G_* cloned in pAT944 | This study |
| pAT973 | 1086-bp XhoI-PstI PCR fragment of truncated *vanR'_G_* cloned in pAT944 | This study |
| pUC1813 | Ap^R^ *lac*Zα vector | [31] |
| pDR111 | Spc^R^, Ap^R^, *lac*I, *amy*E::*P_spank_* | from Rudner |
| pAT948 | EcoRI *cat* cassette cloned into pUC1813 | This study |
| pAT949 | HindIII-SphI fragment of *cat* from pAT948 in pDR111 | This study |

Ap, ampicillin; Cm, chloramphenicol; Fus, fusidic acid; Km, kanamycin; R, resistant; Rif, rifampicin; S, susceptible;

Spc, spectinomycin; Te, teicoplanin; Vm, vancomycin.
