# Supplementary material for: Competition between VanUG Repressor and VanRG Activator Leads to Rheostatic Control of vanG Vancomycin Resistance Operon Expression
Source: PLoS Genet. 2015 Apr 21;11(4):e1005170. doi: 10.1371/journal.pgen.1005170 (PMC4405338; doi:10.1371/journal.pgen.1005170)
Supplement: S2 Table — (DOC) [file pgen.1005170.s006.doc]

**Table S2. Oligonucleotide primers used**

| Primer^a^ | | Sequence (5'->3')^b^ | | Position^c^ | | Restriction site | |
| --- | --- | --- | --- | --- | --- | --- | --- |
| Plasmids |  | |  | |  | |  |
| pG9CAT_NH2_ | GGCTCTAGACAACAAACGAAAATTGGA | |  | | XbaI | |  |
| pG9CAT_COOH_ | GGCTCTAGACCCAAGGCTCCGAGTTGCAGTTA | |  | | XbaI | |  |
| UG1 + | GGTGGTCTCCCATGCGTGTTAGTTATAATA | | 17098-17113 | | BsaI/NcoI | |  |
| UG2 - | CTCCTCGAGACTGTCACGCTCCTTATCGGG | | 17319-17299 | | XhoI | |  |
| RG1 + | GGTGGTCTCCCATGAATGAAAAGATTTTAA | | 17327-17342 | | BsaI/NcoI | |  |
| RG2 - | CTCCTCGAGTTCTTCAATGGTATATCCCAC | | 18028-18008 | | XhoI | |  |
| SG1 + | GGTGGTCTCCCATGAAAGGCTTCACAAAGT | | 18309-18324 | | BsaI/NcoI | |  |
| SG3 - | CTCCTCGAGCGATGAAAGAATCGTCACTTC | | 19148-19128 | | XhoI | |  |
| UG3 + | AAGCTCGAGCAGCTCTGAATCATCAA | | 16576-16592 | | XhoI | |  |
| UG4 - ^d^ | GGCACGCCCGGGTGCTGCCGCCTTCCAGAGCTTATTAT | | 17124-17108 | | SmaI | |  |
| UG5 + ^e^ | GCGGCAGCACCCGGGCGTGCCCCAGAAACCCCCGATAA | | 17290-17306 | | SmaI | |  |
| UG6 - | TTACTGCAGGTGACTGCCTTTCCATA | | 17805-17789 | | PstI | |  |
| RG4 - ^f^ | GGCACGCCCGGGTGCTGCCGCCTCAATTAAATCTGCTA | | 17380-17364 | | SmaI | |  |
| RG5 + ^g^ | GCGGCAGCACCCGGGCGTGCCAAAACCGTATGGGGAGT | | 17993-18009 | | SmaI | |  |
| RG7 - | GTACTGCAGGTGAAGCCTTTCAGATA | | 18319-18303 | | PstI | |  |
| SG4 + | AAGCTCGAGGATTCGGGAACGATATT | | 17524-17540 | | XhoI | |  |
| SG5 - ^h^ | GGCACGCCCGGGTGCTGCCGCCTGGAGCTGTGTATAGT | | 18071-18055 | | SmaI | |  |
| SG6 + ^i^ | GCGGCAGCACCCGGGCGTGCCCGCAGTGAAGATGAAAA | | 19101-19117 | | SmaI | |  |
| SG7 - | TTACTGCAGCGCATTATCTCTGTTGA | | 19617-19601 | | PstI | |  |
| PUG1 + | GGTAAGCTTGCTAGCCAGAGGTGAAAGTTCTTA | | 16901-16918 | | HindIII/NheI | |  |
| PUG2 - | TAAGTCGACCTATGCTTTGCCTATTT | | 17078-17062 | | SalI | |  |
| PYG1 | CCTGCTAGCGCATGGAGGGACAATAA | | 19079-19095 | | NheI | |  |
| PYG2 | TAAGTCGACGCACTCTTGCTTTGACA | | 19256-19240 | | SalI | |  |
| UG_NH2_ + | GGTGGTCTCAAGCTTCATAGT*AAAGAGG*TGGAAACA**ATG**CGT | | 17074-17101 | | BsaI/HindIII | |  |
| UG_COOH_ - | CCTGTCGACGCTAGC*TCA*ACTGTCACGCTCCTT | | 17322-17305 | | SalI/NheI | |  |
| RG_NH2_ + | GGTAAGCTTAGAAGCGTTGCCAGAAA | | 17296-17280 | | HindIII | |  |
| RG_COOH_ - | TAAGTCGACGCTAGC*TCA*TTCTTCAATGGTAT | | 18031-18015 | | SalI/NheI | |  |
| SG_NH2_ + | TTGGCTAGCGTGGGATATACCATTGA | | 18008-18024 | | NheI | |  |
| SG_COOH_ - | TAAGTCGACGCTAGCCCTACGATGAAAGAATC | | 19152-19136 | | SalI/NheI | |  |
| RG10 + | AAGCTCGAGGTCATTACTGGCAGATA | |  | | XhoI | |  |
| RG11 - ^j^ | GGCACGCCCGGGTGCTGCCGCCAGCAAATCAGCTATTT | |  | | SmaI | |  |
| RG12 + ^k^ | GCGGCAGCACCCGGGCGTGCCCCAAGGAAGCCAAAGTT | |  | | SmaI | |  |
| RG13 - | TTACTGCAGCTGATGTTAAAGGCGTT | |  | | PstI | |  |
|  |  | |  | |  | |  |
| T4 terminator |  | |  | |  | |  |
| T4F-HindIII | AGCTTATTATATTACTAATTAATTGGGGACCCTAGAGGTCCCCTTTTTTATTTTAAAAAGCTAGCG | |  | | HindIII/NheI | |  |
| T4R-SalI/NheI | TCGACGCTAGCTTTTTAAAATAAAAAAGGGGACCTCTAGGGTCCCCAATTAATTAGTAATATAATA | |  | | SalI/NheI | |  |
| T4F-NheI | CTAGCGGTACCATTATATTACTAATTAATTGGGGACCCTAGAGGTCCCCTTTTTTATTTTAAAAAG | |  | | NheI/KpnI | |  |
| T4R-NheI | CTAGCTTTTTAAAATAAAAAAGGGGACCTCTAGGGTCCCCAATTAATTAGTAATATAATGGTACCG | |  | | NheI/KpnI | |  |
|  |  | |  | |  | |  |
|  | | |  | |  | |  |
| Labeling of fragments | | |  | | Size of fragment | |  |
| VanG12 + | GCTTGACCTAATTCCAG | | 16826-16842 | | 357 bp | |  |
| VanG126 - | TACTTACTCCAACAGCC | | 17182-17166 | |  | |  |
| VanSG6 + | GCATGGAGGGACAATAA | | 19079-19095 | | 233 bp | |  |
| YG10 - | GTGTTTCATATTCATATGGTT | | 19311-19291 | |  | |  |
| PUG3 | GATATTTATACAATATGCGTT | | 16986-17006 | |  | |  |
| PUG4 | CCTATTTTAGCATACAAAGTA | | 17068-17048 | |  | |  |
| PUG5 | GATATTTATACAATATGCGTTTGATAAAGCT***A***GA***T***TTTGCGAACCAAACACTT | | 16986-17038 | |  | |  |
| PUG6 | CCTATTTTAGCATACAAAGTACGTAAAAGCAAGTGT***A***TG***A***TTCGCAAACTCAAGCTT | | 17068-17012 | |  | |  |
|  |  | |  | |  | |  |
| qRT-PCR |  | |  | |  | |  |
| *vanU_G_* |  | |  | |  | |  |
| VanG129 + | GATAGGGACATGAAAAA | | 17134-17150 | | 188 bp | |  |
| VanG102 - | TCAACTGTCACGCTCCT | | 17322-17306 | |  | |  |
| *vanR_G_* |  | |  | |  | |  |
| VanRG2 + | TGTTACAATAATGCCGC | | 17687-17703 | | 189 bp | |  |
| VanRG10 - | CCTCTGAAGAAATCACT | | 17876-17860 | |  | |  |
| *vanS_G_* |  | |  | |  | |  |
| VanSG2 + | GCAGAGGGTATTCGATA | | 18833-18849 | | 227 bp | |  |
| VanSG10 - | GCAATAGCAAGCCCTAA | | 19060-19044 | |  | |  |
| *vanT_G_* |  | |  | |  | |  |
| VanTG1 + | GCGTATCTGGAAATCAA | | 23884-23900 | | 218 bp | |  |
| VanTG17 - | GCTAGAGATGCCATAT | | 24102-24087 | |  | |  |
| *rpoB* (AE016830) | | |  | |  | |  |
| rpoB5 + | CGTGACGTTCACTACTCT | |  | | 198 bp | |  |
| rpoB12 - | CTTCGATGTCTGCTGTT | |  | |  | |  |

^a^ +, sense primer; antisense primer.

^b^ Restriction sites incorporated into the oligonucleotide are underlined; in UG_NH2_, the RBS introduced is in italics and underlined and the initiation codon is in bold and underlined; the stop codon in UG_COOH_ and in RG_COOH_ is in italics; mutations in PUG5 and PUG6 are in italics and in bold.

^c^The nucleotide numbering of *E. faecalis* BM4518 was used (GenBank accession number AY271782).

^d^ UG4, 10 first amino acids remain at the N-terminal part of VanU_G_.

^e^ UG5, 11 last amino acids remain at the C-terminal end of VanU_G_.

^f^ RG4, 19 first amino acids remain at the N-terminal part of VanR_G_.

^g^ RG5, 13 last amino acids remain at the C-terminal end of VanR_G_.

^h^ SG5, 9 first amino acids remain at the N-terminal part of VanS_G_.

^i^ SG6, 16 last amino acids remain at the C-terminal end of VanS_G_.

^j^ RG11, 18 first amino acids remain at the N-terminal end of VanR'_G_.

^k^ RG12 19 last amino acids remain at the C-terminal end of VanR'_G_.
